# Going, going, gone: competitive decision-making in Dutch auctions

**DOI:** 10.1186/s41235-020-00259-w

**Published:** 2020-11-30

**Authors:** Murray Bennett, Rachel Mullard, Marc T. P. Adam, Mark Steyvers, Scott Brown, Ami Eidels

**Affiliations:** 1 School of Psychology, The University of Newcastle, Callaghan, NSW 2308 Australia; 2 School of Electrical Engineering and Computing, University of Newcastle, Callaghan, Australia; 3 Department of Cognitive Sciences, University of California, Irvine, Irvine, USA

**Keywords:** Dutch auction, Group decision making, Competitive, Prospect theory

## Abstract

In a Dutch auction, an item is offered for sale at a set maximum price. The price is then gradually lowered over a fixed interval of time until a bid is made, securing the item for the bidder at the current price. Bidders must trade-off between certainty and price: bid early to secure the item and you pay a premium; bid later at a lower price but risk losing to another bidder. These properties of Dutch auctions provide new opportunities to study competitive decision-making in a group setting. We developed a novel computerised Dutch auction platform and conducted a set of experiments manipulating volatility (fixed vs varied number of items for sale) and price reduction interval rate (step-rate). Triplets of participants ($N=66$) competed with hypothetical funds against each other. We report null effects of step-rate and volatility on bidding behaviour. We developed a novel adaptation of prospect theory to account for group bidding behaviour by balancing certainty and subjective expected utility. We show the model is sensitive to variation in auction starting price and can predict the associated changes in group bid prices that were observed in our data.

People behave differently in the presence of others than when they are on their own. Social psychologists, for example, have documented phenomena such as social facilitation (Zajonc 1965)—the tendency to perform better in simple, well rehearsed tasks than when in the presence of others (e.g. Lee et al. 2011; Lichtendahl et al. 2013; Kvam 2018). In the current study, we investigate the effect of group context on human decision making, and in particular how the presence of other competitors affects bidding behaviour in a computerised Dutch-Auction task.

A *Dutch auction* is a descending price auction where an item begins at a set maximum price. The price is gradually lowered over a fixed period of time until a bid is placed that guarantees the bidder the purchase of the item at the current price (Thomas 2012). Decision-making behaviour in a Dutch auction requires participants to balance the speed of response (i.e. time of bid) with the consequence of that response (i.e. price). Bidding earlier at a price equal to or greater than the value of the item increases the probability of winning but also of overpaying for the item, which would result in no resale profit (Vickrey 1961). On the other hand, the longer a bidder delays in placing a bid the higher the likelihood the bidder increases their profit on resale, but the risk of losing to a competitor also increases. The way in which a bidder trades-off between the certainty of winning and the price they pay is the key to developing a bidding strategy within a Dutch auction (Easley et al. 2010) and makes for an ideal context for the study of competitive decision making.

There were three aims of the present study. The first aim was methodological, to develop a computerised platform for the study of human decision making in Dutch auctions. This context is particularly interesting as it allows the examination of human behaviour in a group, within a relatively controlled environment in which people can trade-off certainty and value.

The second aim was a practical one. Dutch auctions are a real commercial vehicle, running upward of 400 million USD in revenue from the Dutch flower auctions alone in 2018 (Royal-FloraHolland 2018). Stakeholders (auction houses, potential auction participants) may wish to know how key factors in the auction set-up affect the behaviour of bidders. For example, faster clock speeds (i.e. faster rate of decrease) that result in short duration auctions yield lower prices compared to slower clock speeds with longer duration (Katok and Kwasnica 2008). A review of the Dutch auction literature by Adam et al. (2017) revealed an apparent gap in Dutch auction experiments in which the *perceived* difference in step-time had not been examined. This issue has important practical implications: for a given price, and a given rate of price change, the subjective experience of the auction participants and their tendency to bid could vary with larger or smaller price steps. Auction houses and bidders alike should know whether manipulations of price step would affect their prospect. Using hypothetical funds, we examined the effect of continuous versus discrete changes in price on bidding behaviour, while keeping overall decrease rate fixed (practically, price declined over ten slow steps, or one hundred shorter steps with a small price change in each). We also examined the effect of volatility, by presenting participants a fixed amount of items for sale in each auction (Experiment 1) or a variable amount (Experiment 2). We employed Bayesian analysis that circumvents limitations of commonly used frequentist tests and allows direct tests of the null hypothesis.

The final aim was theoretical. Prospect theory (Tversky and Kahneman 1992) has been widely considered in ascending price auction formats; however, it has not been applied to Dutch auctions in a quantitative manner. We begin a preliminary framework for a dynamic extension of prospect theory to account for the way multiple bidders in Dutch auctions trade-off between the certainty of winning and the subjective utility of the items for sale.

The Dutch auction originally obtained its name from local arts and flower markets in the Netherlands (Adam et al. 2017). With perishable goods, such as flowers, sellers must limit the auction duration to ensure the goods are fresh. For readers unfamiliar with considerations affecting bidding behaviour in Dutch auction, the following illustrative example could be helpful. Suppose you are a florist and have a limited budget with which to restock your shop for the week. To do this, you attend a Dutch auction where you compete with other florists to purchase flowers from a limited pool of flowers. To fully restock your shop using your limited budget, you must determine what bids to place that allows you to purchase enough flowers at the lowest possible price. To purchase your flowers for the lowest price, it would be best to wait as long as possible before making a bid. However, by waiting you risk losing the flowers to another florist who might bid earlier. So, for a better chance of purchasing enough stock to fill your shop it would be better to bid early. Yet, by doing so you pay a higher price, potentially depleting your limited budget before you have obtained enough flowers to restock your shop. Therefore, to obtain enough flowers for your shop using a limited budget you must balance the price you are prepared to pay against the risk of losing to a competing florist to determine the best possible bidding strategy.

The example demonstrates how decision-making behaviour in a Dutch auction requires participants to balance the speed of response (i.e. time of bid) with the consequence of that response (i.e. price). Despite the popularity of auctions in general, limited research has been done on Dutch auctions compared to other auction formats (Adam et al. 2017). Moreover, the perspective of these investigations had also been limited: to date, much of the research on Dutch auction has been conducted in economics with the focus of identifying conditions that yield optimal price. It is not entirely clear how various factors affect bidding behaviour and in particular the trade-off between price and speed (or timing) of bidding in the Dutch auction.

In the following sections, we discuss the trade-off between certainty and price in developing bidding strategies in Dutch auctions. We then discuss the effect of competitors and clock speed (the rate of price decline) on bidding behaviours. After surveying these factors, we describe our novel computerised platform and report data from two laboratory experiments. Using Bayesian statistics, we show that contextual factors of discrete and continuous changes in price and stock volatility have little effect on the behaviour of bidders. Finally, we present a dynamic extension of prospect theory (Tversky and Kahneman 1992) to account for the way bidders trade-off certainty with subjective utility. We show the theory can capture major trends in data.

## Certainty versus price: effect of certainty on bidding behaviours

Bidders in Dutch auction must decide whether to prioritise the certainty of winning or the price they are willing to pay. A fundamental and unique feature of this auction format is that the first bidder wins the available item with certainty (Turocy et al. 2007). To gain certainty in the context of a Dutch auction, a winning bid must be placed earlier and at a higher price. Ample evidence suggests people prefer certainty over uncertainty in a variety of conditions (e.g. Kahneman and Tversky 1979, 2013).

Certainty matters; bidders may prioritise certainty of winning in an attempt to avoid the negative emotional state caused by losing. Adam et al. (2012) found participants felt the loss of a Dutch auction more strongly than a win, and suggested participants were better able to cognitively prepare for the win, as, in placing a bid, they expected certainty in obtaining the item. A loss, on the other hand, was unexpected causing a greater emotional response.

Prioritising certainty helps bidders avoid negative emotions associated with losing, but also increases the risk of falling victim to the winner’s curse. The winner’s curse is the potential for the winning bid to exceed the value of the item being auctioned (Easley et al. 2010). This phenomenon is prevalent in common value auctions, where the value of the item is the same for all bidders, but bidders do not know this value and thus base their bids on their own information and private valuations (Kagel and Levin 1986). This leads the bidder with the highest estimation of the item’s true value to win the auction for an exorbitant value (hence the term ‘winner’s *curse*’) (Van Den Bos et al. 2008). Garvin and Kagel (1994) found that bidders adapt their bidding strategies to reduce the winner’s curse. They showed that while inexperienced bidders initially had high rates of the winner’s curse, these bidders could learn to reduce the effect through feedback. Furthermore, they found this learning process was aided through the observations of the winner’s curse in other bidders. This suggests that an individual’s bidding behaviour is a dynamic and malleable process that can be continually adjusted through their bidding experience and through their opponents bidding experience.

However, while this study showed that bidders can adjust their bidding strategy through learning, the effect may not be as influential as first thought. Indeed, Lind and Plott (1991) found the winner’s curse was still present in experienced bidders albeit at a reduced magnitude. In the present study, we report simple block-by-block analysis of group averaged bids to examine possible effects of learning on bidding and in particular if and how bidding behaviour changes in the course of a testing session.

## Effect of competitors on bidding behaviour

The presence and behaviour of other competitors can have a significant impact on bidding behaviour, particularly in the way a bidder trades-off between certainty and price. An investigation by Teubner et al. (2015) on the effect of computerised bidders on human bidders in first price sealed bid (FPSB) electronic auctions showed participants’ overall emotional arousal and bids were lower when competing against computerised bidders, compared to human bidders. Teubner et al. (2015) suggested there is a socially competitive nature to auctions, where participants gain greater satisfaction in winning against other humans compared to computer opponents. Cassady (1967) found that bidders in a Dutch auction experience as much, if not more, competitive pressure compared to other auction formats. With the increased certainty of winning in Dutch auctions, there also comes an increased risk of an immediate and certain *loss* to another competitor. This risk may have a range of effects on how bidders determine the trade-off between certainty and price. Ku et al. (2005) proposed that the level of perceived competition could result in an intense emotional state of *competitive arousal* which reduces an individual’s ability to think clearly and increases risk-taking behaviours. Adam et al. (2012) found that greater competitive arousal led participants to delay their bid, particularly during faster Dutch auctions, thus increasing the risk of losing the auction to a competitor but increasing the chance of a higher nominal payoff. How bidders perceive the level of competition can also be affected by the available information regarding competitors. A bidder must account for the strategies of other competitors by using any information they may have on the likely bids of their competitors (Vickrey 1961), which in turn will be based on the competitor’s beliefs about the original bidder’s strategies (Rafaeli and Noy 2005).

In multi-unit Dutch auction experiments, many homogeneous items are offered for sale within the same auction. The first to bid identifies the number of items they wish to purchase at that price, and the auction continues with the remaining items till all are sold (Mochón et al. 2015). Buchanan et al. (2016) observed a reduction in bid prices during multi-unit Dutch auctions when there was greater information available to bidders regarding their competitors, such as the number of participating bidders or the total remaining items, compared to when there was no information available to participants.

In contrast to multi-unit auction, in a one-off Dutch auction the auction ends with the first bid, so a bidder cannot base their bid on the behaviour of competitors and instead must rely on other sources of information (Nakajima 2011). In the Dutch auction context, bidders compete against each other over consecutive auctions so it is possible to gain information about competitors’ bidding behaviours through repeated observations. The design of the current study allowed participants to compete against each other over multiple trials (i.e. auctions), such that they could form a view of competitors’ bidding strategies and adjust their behaviour accordingly. An appreciation of competitors’ strategy requires learning over time and should be visible by examining changes in bidding behaviour across testing blocks, which we have tested in Experiments 1 and 2.

Research to-date shows that the desire to win, heightened by competitive arousal, and time pressures associated with auctions have a significant impact on bidding behaviours (Malhotra et al. 2008). This often causes bidders to focus on beating their opponents rather than maximising their profit, resulting in overbidding (Malhotra et al. 2008). Note though that the aforementioned studies focused on ascending auction formats, and it is yet unknown whether and to what extent these effects come into play in *descending* auctions such as Dutch auctions.

## Effect of clock speed on bidding behaviour

*Certainty* in Dutch auctions concerns not only the identity of the winner, but also certainty in ending the auction itself. Thus, bidders have the ability to control the duration of the Dutch auction, which in turn allows bidders the ability to trade-off between time saved and price. If a bidder prioritises time, they may place an earlier bid. This would result in a higher price but at the same time (pardon the pun) also enables bidders to save time in auction participation. Lucking-Reiley (1999) proposed that bidders may neglect optimal bidding strategy and save time by terminating auctions prematurely after finding higher revenues in Dutch auctions compared to first-price sealed-bid auctions. In first-priced sealed-bid auctions, bidders submit a bid by a set time, after which the bids are examined and the highest bid wins the item. Lucking-Reiley (1999) suggested that bidders in the Dutch auction may become impatient due to extended auction periods (i.e. days) resulting in early bids to end the auction quickly. Katok and Kwasnica (2008) examined systematic manipulations of the auction clock in a controlled laboratory setting by controlling the time intervals with which the price changed (i.e. 1, 10 and 30 s). They maintained a constant start price of 100 tokens and a fixed-size price decrements of 5 tokens. Thus, to illustrate, in the 1 second condition price would start at 100 tokens, then reduce to 95 tokens after one second and then to 90 after another second, whereas in the 30 s condition price again starts at 100 tokens and remains fixed for 30secs, at which point it drops to 95 and so on. They found that the speed of the Dutch auction clock significantly affected the price of the winning bid in the slow condition (30sec) resulting in higher prices compared to the fast condition (1sec). Similar to Lucking-Reiley (1999), they proposed that bidders view time as a valuable resource causing impatient bidders to bid early—and thus at higher prices—in order to end a slow Dutch auction early and save time.

The current study extends the work of Katok and Kwasnica (2008) by examining the effect of different patterns of price change, where the duration of the auction is held constant but the size of the price decrements changes (small and large), creating what looks like continuous and discrete patterns of price changes, respectively. The continuous price condition introduces small decrements in price quickly, over small time steps, resulting in what is perceived to be as a smooth change in price. The discrete price condition introduces large decrements in prices, over relatively long time steps, resulting in perceived jumps in price.

## Developing a computerised platform for testing Dutch-auction behaviour

In developing our testing platform, we aimed to design a computerised Dutch auction program that enables researchers to manipulate a range of design parameters that have been found to affect bidding behaviours (see Adam et al. 2017). Experiment code for the platform is freely available at: https://osf.io/jyv3u/. Cox et al. (1982) identified three fundamental design parameters that affect bidding behaviours in Dutch auctions. First, the starting price of the available commodities needs to be set at a value greater than that of the highest price any bidder is willing to pay. This is normally at a value greater than the value of the commodities for sale. Second, there needs to be a set time between each price decrease. Within Dutch auctions, this is reflected in the tick of the auction clock. Finally, there needs to be a set value by which the price decreases at each time point. The set time between price decreases and value of price decreases determines the speed of the Dutch auction clock. Any platform designed for testing behaviour in Dutch auction experiments must satisfy these criteria.

Another important consideration in platform development was the parameter unit quantity (the amount of items presented on every sale). Research has shown information related to unit quantity can influence bidding behaviour in Dutch auctions (Buchanan et al. 2016). To address this, we included a design feature that enables the unit quantity to be fixed or varied. This feature not only allows this platform the potential to be used in both single-unit or multi-unit studies but also allows for further research into the effect of unit quantity information. With a *fixed* unit quantity, bidders are provided certainty in the number of available units available in each auction while in a *varied* unit quantity condition the number of units available on each auction is unknown, creating additional uncertainty through the volatility in the bidding environment.

Another important design aspect was performance feedback. Garvin and Kagel (1994) highlighted bidder’s ability to adjust behaviours through both experience and observations. We therefore included a design feature that provides participants with information relating to their performance.^1^ This feature allows future investigations to examine effects of learning on bidding behaviour in Dutch auctions. Finally, we integrated within the platform an option for either human players or a computer competitor. When used in experimental settings, this would allow researchers to compare bidding behaviour when competing against a computer model and when competing against other human bidders. Constructing a suitable computer competitor model is a serious challenge and is well out of the scope of the current study. Although we do not report results from a human–computer competition in the current report, building this feature into our platform will allow future researchers to develop suitable computer models, and easily integrate them in the Dutch auction context.

## Overview of the current study

The practical goals of the present study were to develop a computerised testing platform for Dutch auction and to examine competitive decision making in this group context. The theoretical goal was to develop the first quantitative theory for bidding behaviour. Using the new testing platform, we conducted two in-lab experiments that investigated the effect of different patterns of price changes and bidding experience on bidding behaviours within a computerised Dutch auction. To investigate the effect of different patterns of price changes, we examined the dependent variables of Price of the winning bid and Step (i.e. time at which participants bid) of winning bid using hypothetical funds in two different price change conditions (discrete vs continuous). Although this was not a main goal, we examined the potential effect of experience by considering bidding price and step over testing blocks. Finally, we examined the relationship between starting price and winning bids. Some auction studies show lower starting prices lead to higher bids (e.g. Ku et al. 2006), while other studies suggest the opposite (lower start prices lead to lower bids (e.g. Ariely and Simonson 2003). We tested this relationship by calculating the correlation between auction start price and winning-bid values and by comparing winning bids on auctions with low ($50–100) and high ($100–150) start prices.

Triplets of participants competed in two versions of a computerised Dutch auction. In Experiment 1 (“Fixed”), they were allocated hypothetical funds to bid on a fixed amount of stock available on each trial to fill a virtual warehouse. Participants played the game in a continuous price change condition, where the price of the stock decreased every 50 ms, and in a discrete price change condition where the price of the stock decreased every 500 ms. Experiment 2 (“Variable”) was similar to Experiment 1, except that the amount of stock available on each trial varied randomly (and uniformly) between 50 and 150 units.

To ensure they could stock their warehouse within their limited budget, participants had to determine what bids to place. By bidding early, they could gain an increased degree of certainty in winning the stock but pay a higher price, increasing the risk of depleting their limited funds before they filled their warehouse. In delaying their bids, they could purchase the stock at a lower price but also increase the risk of losing the stock to a competitor.

## Experiment 1: fixed-unit

### Method

Participants competed in a computer simulated Dutch auction game in three-person competitive groups over the two experimental phases. Testing was done in-lab, with all three participants seated in the same room. Participants were asked to use a set allocation of hypothetical funds to purchase a *fixed* amount of stock to fill a virtual warehouse.

#### Participants

Thirty-three students ($$M_{\mathrm{age}} = 23.3, {\hbox {SD}}_{\mathrm{age}} = 6.1$$; 15 females and 18 males) from the University of Newcastle, Australia, completed the experiment in 11 three-person groups in exchange for a $25 shopping voucher or course credit, depending on sign-up source. All participants reported English as their first language and had normal or corrected to normal vision, including colour vision. This study was approved by the University of Newcastle Human Ethics Research committee.

#### Stimuli and design

The task was separated into two conditions of decreasing price rate: *continuous steps* and *discrete steps*. Continuous price decrements occurred every 50 ms over a 5000 ms trial, for a maximum of 100 price changes. The discrete price decrements occurred every 500 ms, resulting in 10 price changes over a 5000 ms trial. We recorded (1) the price of the winning bid and (2) the step at which the winning bid was made.

Both tasks used a computerised Dutch auction presented online in Javascript. Tasks were completed over 1 practice block and 5 experimental blocks each consisting of 12 trials. On each trial, the price of the items for sale was continually lowered until a bid was made, guaranteeing the bidder the purchase of the items for the current standing price.

**Fig. 1:**
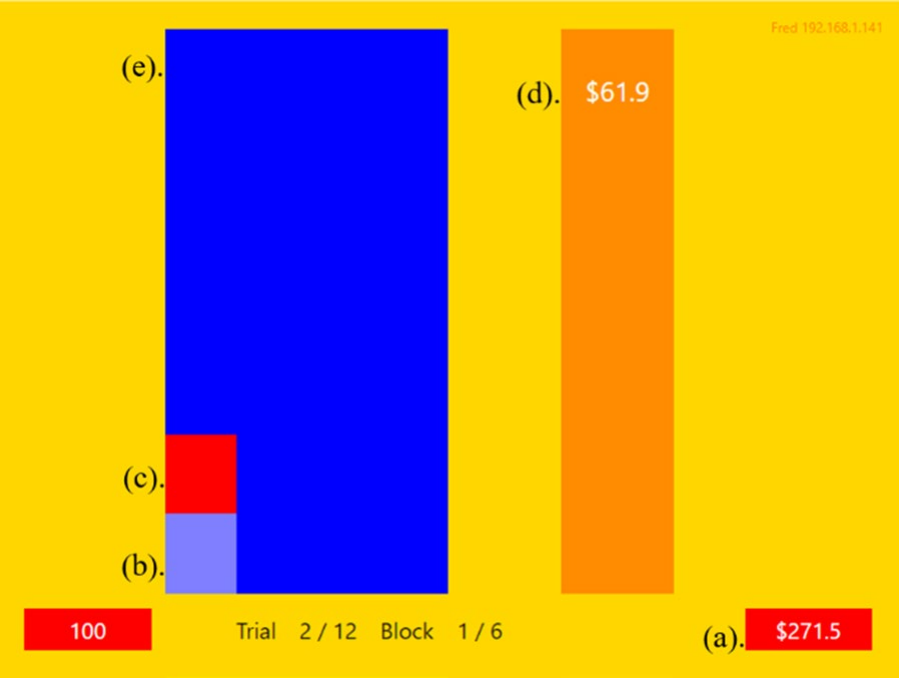
Example of the game interface. **a** Participants available funds, **b** participants current warehouse stock, **c** available stock for purchase, **d** available stock sale price, **e** participants virtual warehouse

Participants completed the experiment in the Newcastle Cognition Lab on DELL OptiPlex 7040 desktop computers with a 23-inch DELL P2314H monitor. Each participant interacted with their own game interface, a $16.9 \times 12.7$ cm rectangle (representing a visual angle of $16.03^{\,\circ }$ and $12.08^{\,\circ }$, respectively) , as shown in Fig. 1. The game interface included player’s available funds (Fig. 1a) displayed in white text in a $2.4 \times 0.8$ cm box (visual angle of $2.29^{\,\circ }$ and $0.76^{\,\circ }$, respectively) , current warehouse stock displayed in light blue (Fig. 1b), stock available for purchase (Fig. 1c) displayed as a red bar atop the current stock, sale price of the available stock (Fig. 1d) presented in white text over a 2.1 $\times$ 10.6 cm ($2.00^{\,\circ }$ and $10.09^{\,\circ }$) orange box, and the players warehouse (Fig. 1e) displayed in a dark blue 5.3 $\times$ 10.6 cm ($5.06^{\,\circ }$ and $10.09^{\,\circ }$) box.

The decreasing price of the available stock in each trial was represented visually via the price bar descending in tandem with the numerical countdown represented by the text value. The starting price of each trial was randomly sampled from a uniform distribution between $50 and $150. The available stock was fixed at 100 units for each trial. All players could bid to purchase the available stock at any time throughout the trial.

**Fig. 2:**
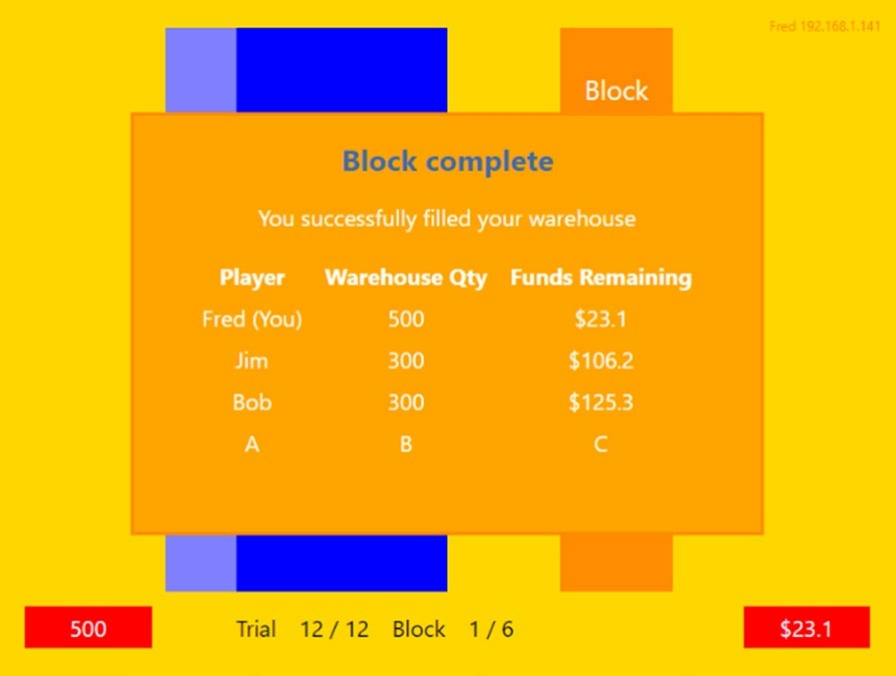
Example of the feedback provided to participants at the completion of each block. Participants were randomly assigned pseudonyms for the duration of the experiment

Participants were allocated a hypothetical amount of $250 and a warehouse capacity of 500 units. Trials were completed when a bid was placed or the descending price bar and associated value reached $0. A successful bid was indicated to the player by the price bar changing colour to green and the red available-stock block changing colour to light blue. Unsuccessful players were informed of the winning bid by their price bar changing colour to red and the available stock bar disappearing. Players warehouses were deemed full once the light blue block reached the top of the dark blue box at which point they could no longer bid. A feedback screen (Fig. 2) following the completion of each block informed participants of the groups performance and their own by listing the remaining funds and total stock obtained for each participant.

**Fig. 3:**
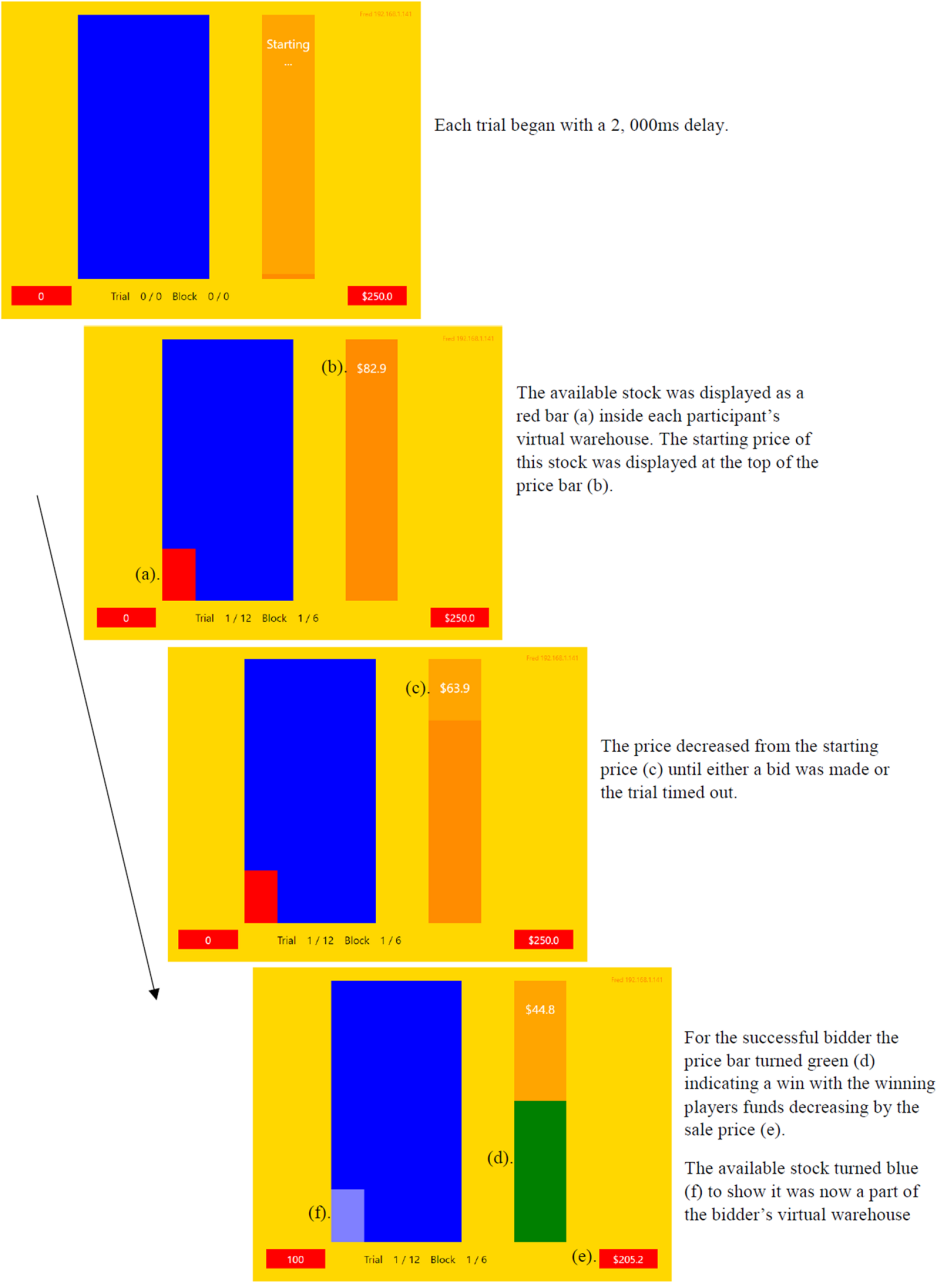
Example of display sequence within a single auction trial

#### Procedure

Participants completed the experiment in the cognitive laboratory at the University of Newcastle in a well-lit testing room with four computers—one per player and a control computer for the experimenters. Participants were presented an information statement and provided written consent before answering demographic questions regarding age, gender, handedness and vision. Participants were then placed into groups of three and presented with instructions explaining the step-rate phases of the experiment. Participants were asked to imagine they were a store manager who needed to restock their warehouse and to do so were required to participate in a computer simulated Dutch auction. Participant groups were pseudo-randomly allocated to complete the discrete or continuous conditions during the first phase.

The experiment began when all participants indicated they had read and understood the instructions. Participants completed one practice block followed by 5 testing blocks of 12 trials for each of the four conditions. Figure 3 illustrates the sequence of events within a single auction trial. The number of units available to competitors was fixed at 100 units for every trial. Participants placed their bid by pressing the spacebar on their keyboard. Each trial ran for 5000 ms with the price of available stock decreasing from the starting price to $0 over this time period. Trials began following a 2000 ms delay. Participant funds and warehouse capacity refreshed at the beginning of each block. The experiment concluded following the completion of all conditions over approximately 30 min.

### Results and discussion

Practice block data were removed from analysis. “Step” data from the discrete-step condition was normalised via multiplication by 11 to allow comparison to the continuous-step data. We report results using both frequentist tests and Bayes factors to allow the quantification of evidence supporting the null or alternate hypothesis. We provide a summary of the primary analyses in Table 1. Price and step of the winning bid were assessed as the aggregate *group* bid or step. We report the mean performance of the group rather than each individual across the different conditions, since the winning bids for the group are always known, whereas individual players’ bids are known only on one third of auctions, on average (only those auctions in which they won).

**Table 1:** Experiment 1 results summary

		Price		Step	
		*p*	${\hbox {BF}}_{10}$	*p*	${\hbox {BF}}_{10}$
Group (1–11)	Continuous	.49	.003	.06	.07
	Discrete	.99	< .001	.99	< .001
Continuous versus discrete	Means	.06	.05	.87	.04
Block (1–5)	Step rate	.69	.23	.92	.23
	Block	.56	.09	.26	.21

*Denotes significance

#### Relationship between price and step

**Fig. 4:**
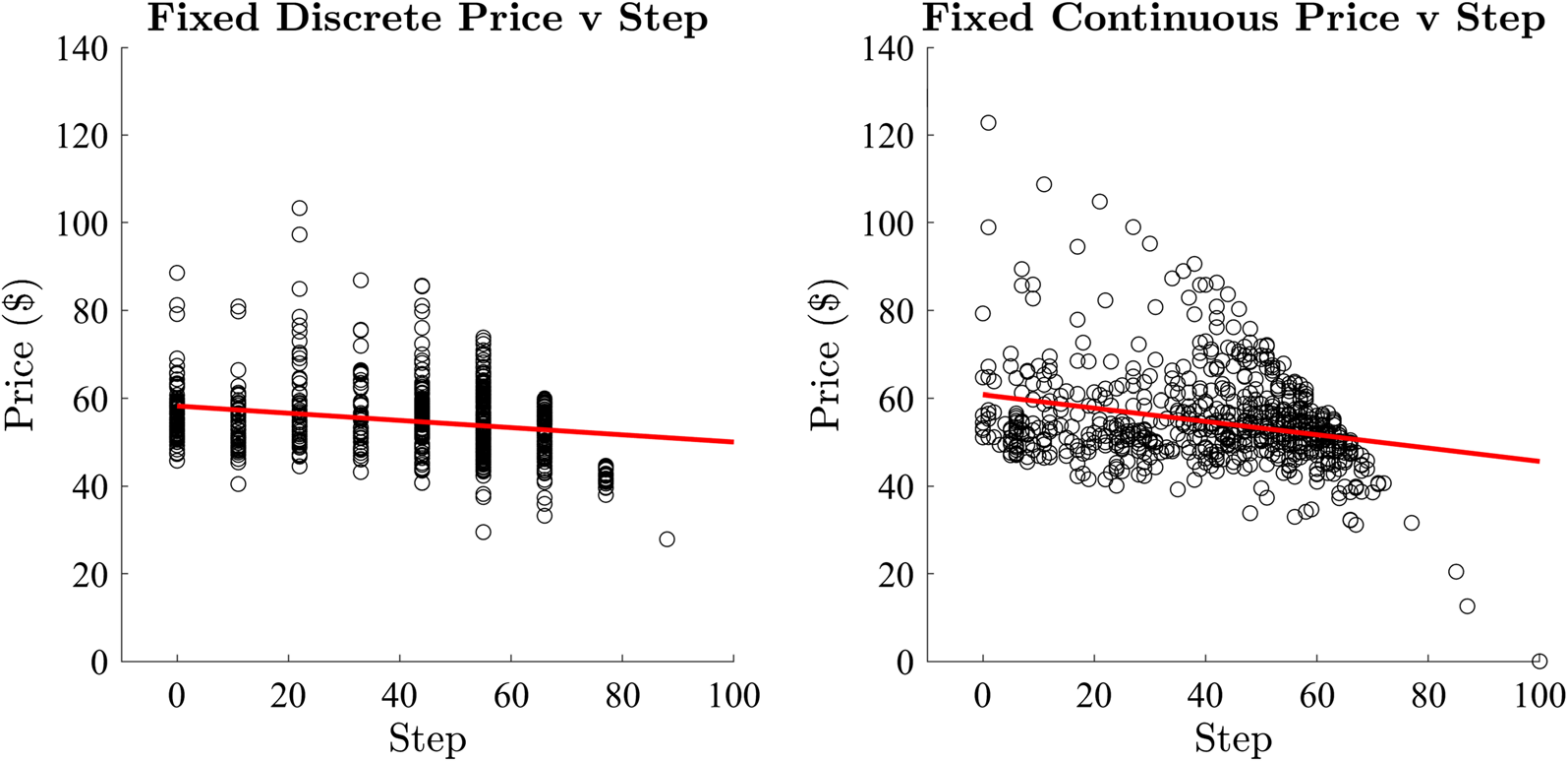
Exp 1: price and step correlation

The two dependent variables in this study, *Price* and *Step* of the winning bid, are related by design (i.e. price decreases with each step). However, the starting price on each trial was stochastic, where the initial value varied uniformly between $50 and $150, yet the step was deterministic [the step always decreased from 10 (or 100) to 1]. To demonstrate the possible effects on bidding behaviour consider two trials where the winning bid is approximately $50). If the first trial has a starting price of $150, then participants must wait for a late step before the price reaches $50. Whereas on the second trial the starting price is $60 so participants may bid early, if not immediately. Thus, the price and time of bids should be related (negatively: price goes down with time) but should not be perfectly correlated. To understand this relationship, we calculated the correlation between the price and the step of the winning bid for both the discrete and continuous price change conditions to examine this relationship (Fig. 4). There was a significant moderate negative correlation between price and step in the discrete price-change condition ($$r = -0.23, R^2 = 0.05, p < 0.001$$). There was also a significant moderate negative correlation between price and step in the continuous price-change condition ($$r = -0.27, R^2 = 0.07, p < 0.001$$). Given that price and step of the winning bid are related, but are not perfectly correlated, we examine the data using both of these measures.

#### Effect of participant group

**Fig. 5:**
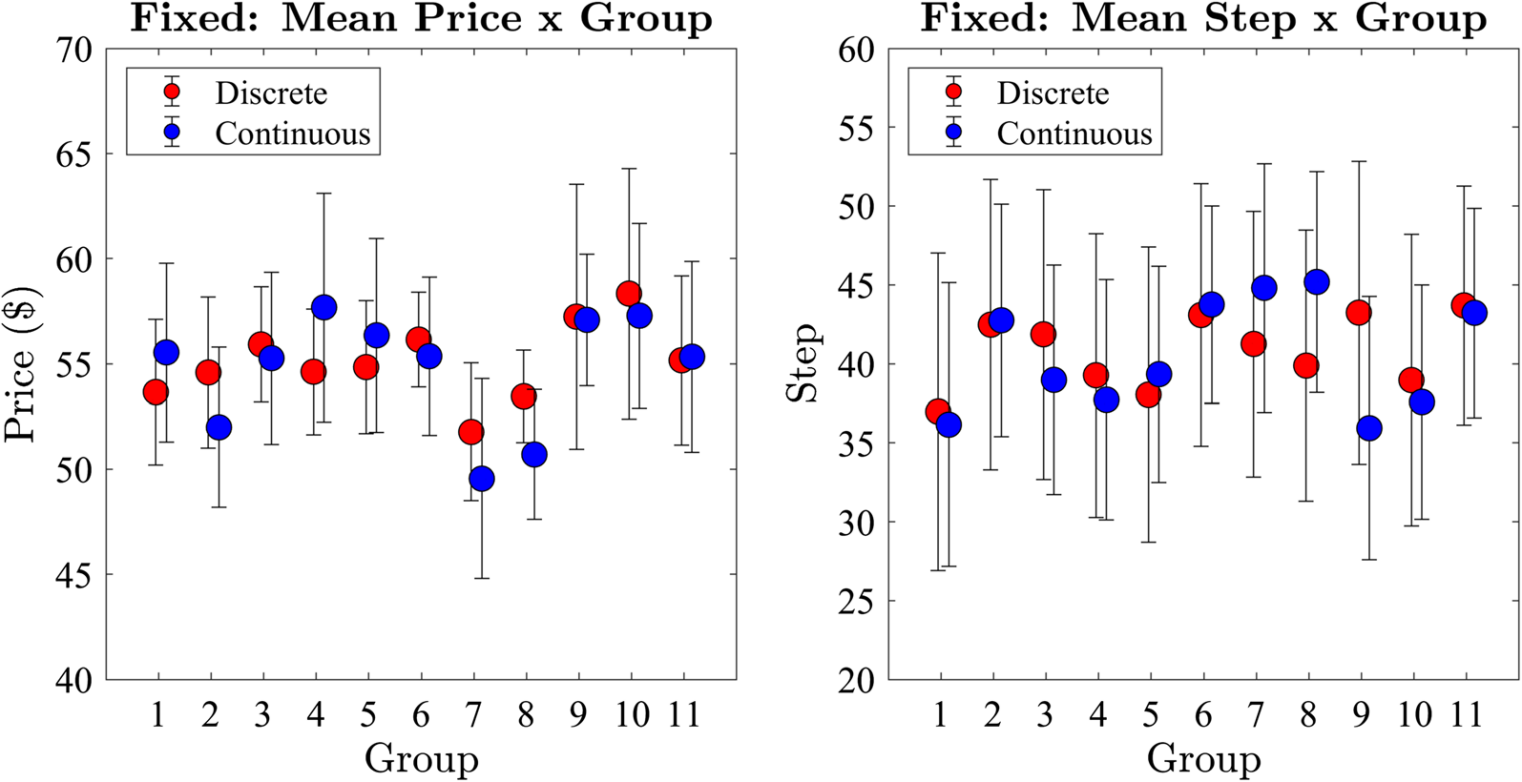
Exp 1: mean price and step per group

We began by examining effects between participant groups on the price and step of the winning bid (Fig. 5). We conducted a one-way between subjects ANOVA with the price the winning bid for the discrete and continuous step conditions. We found that the price of the winning bid was not significantly different between participant groups in either the discrete ($F(10,649) = .27, p = 0.99, BF_{10} < 0.001$) or continuous ($F(10,649) = .95, p = 0.49, BF_{10} = 0.003$) conditions. We repeated the analysis with the step of the winning bid and also found no significant effect of participant group on the step of the winning bid in either the discrete ($F(10,649) = .27, p = 0.99, BF_{10} < 0.001$) or continuous ($F(10,649) = 1.8, p = 0.06, BF_{10} = 0.07$) conditions. We collapsed the price and step data across groups for further analysis given these outcomes.

#### Discrete versus continuous price change

We next compared the price of the winning bid between the discrete and continuous price change conditions. The assumption of normality was violated ($W = 0.93, p < 0.001$), so we utilised the nonparametric Wilcoxon signed rank test. Participant bids were not significantly different ($W = 99,673, p = 0.06, BF_{10} = 0.05$) when bidding in the continuous ($M = 54.97, SD = 3.98$) or discrete ($M = 54.71, SD = 3.47$) price change conditions.

**Fig. 6:**
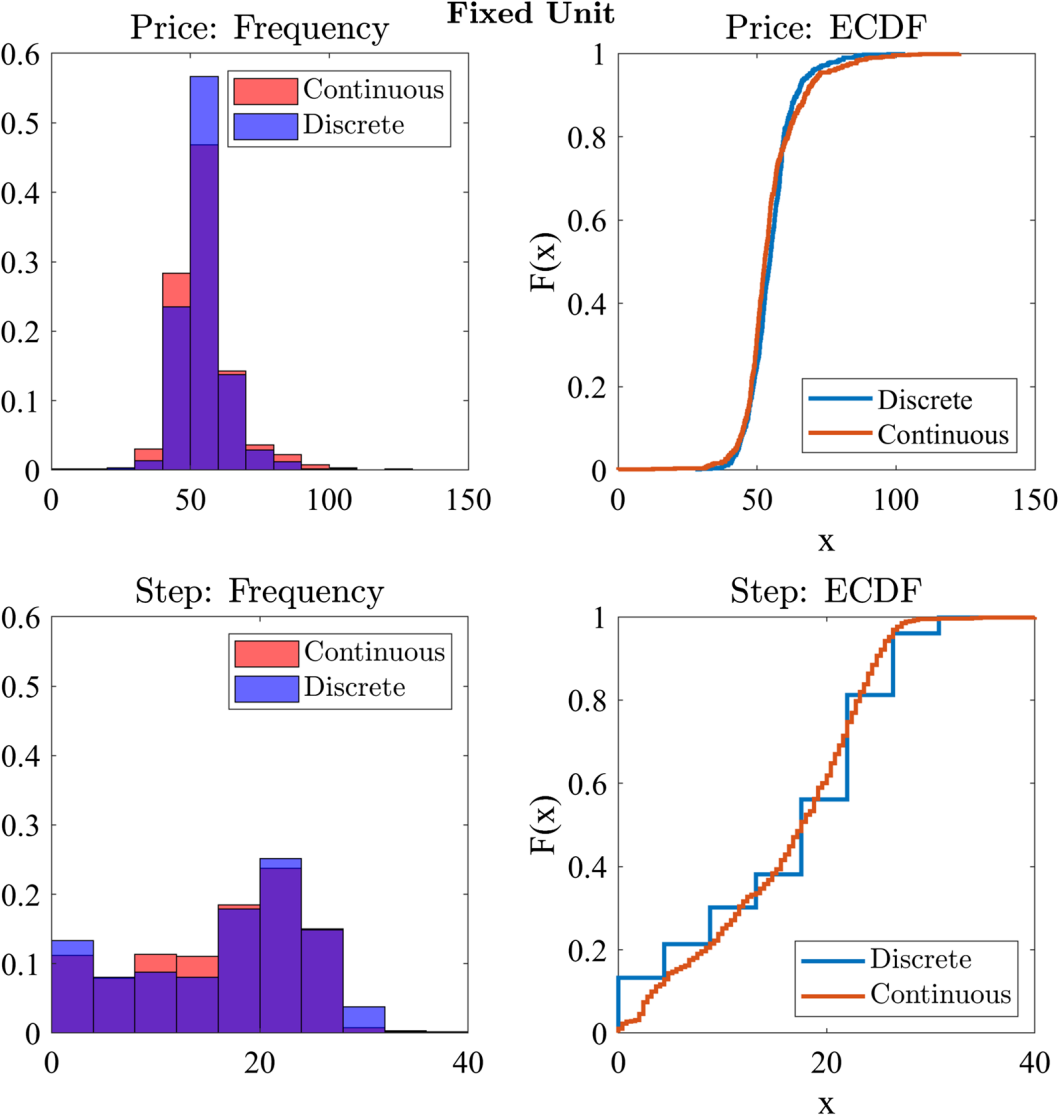
Frequency distributions and empirical cumulative distribution functions for price and step of the winning bid across continuous and discrete price change conditions

We repeated the Wilcoxon signed rank test analysis on the step of the winning bid and found that participants did not significantly differ ($W = 103,076, p = 0.87, BF_{10} = 0.04$) on the step of the winning bid between the continuous ($M = 40.01, SD = 5.78$) and discrete ($M = 40.15, SD = 5.66$) step conditions (Fig. 6).

#### Effect of block

**Fig. 7:**
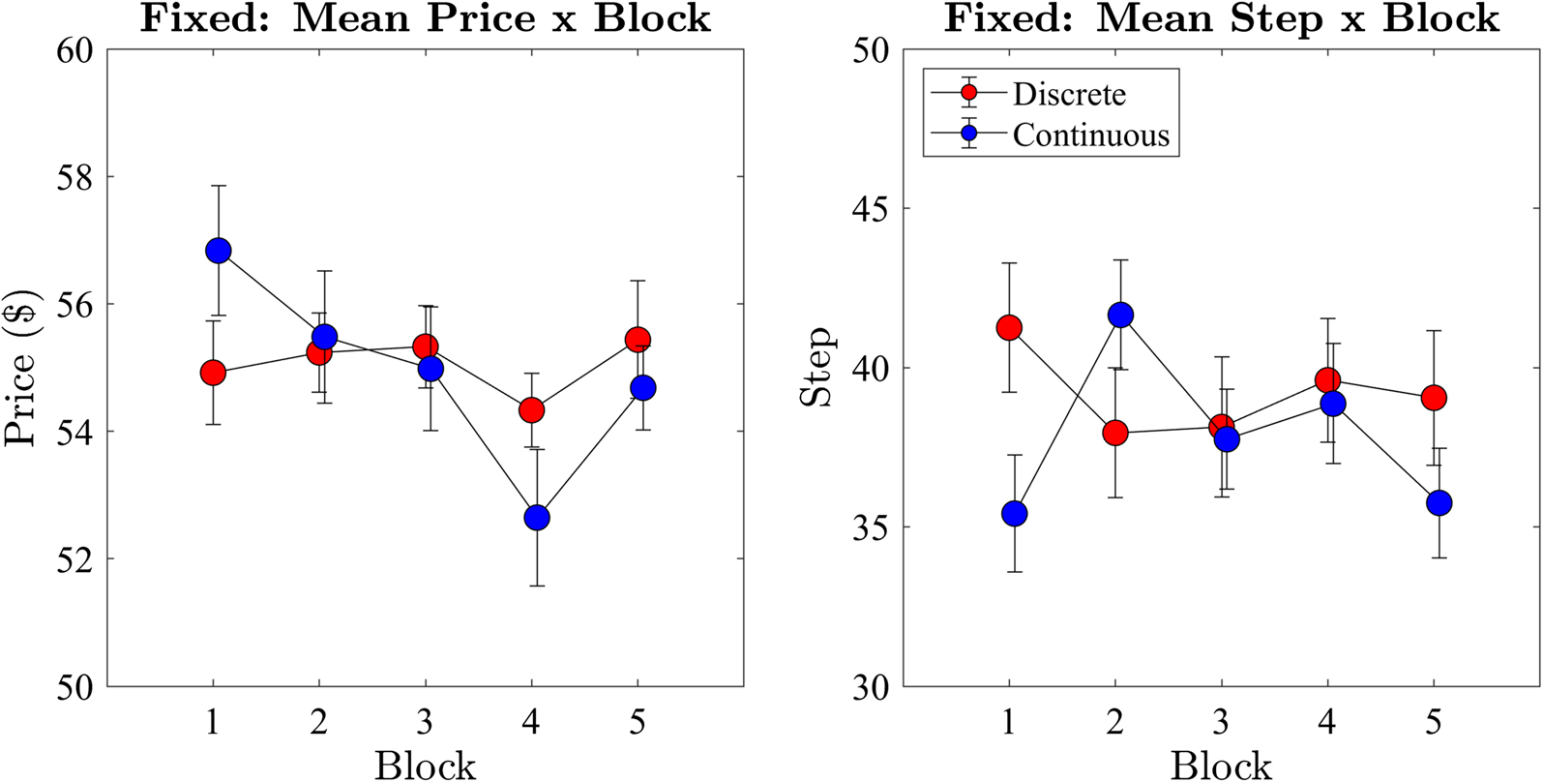
Exp 1: mean price and step per block

We examined the price of the winning bid across block progression (Fig. 7) using a two-way ANOVA with within subjects factors of step-rate (discrete and continuous) and block (1st-5th). We found no significant difference ($F(1,10) = .17, p = 0.69, BF_{10} = 0.23$) in the price of the winning bid between the continuous ($M = 54.7, SD = 10.87$) and discrete ($M = 55.0, SD = 8.37$) conditions. There was also no main effect of block ($F(4,40) = .75, p = 0.56, BF_{10} = 0.09$) on the price of the winning bid.

We repeated the ANOVA with the step of the winning bid using the same factors. We found no significant difference ($F(1,10) = .011, p = 0.92, BF_{10} = 0.2$) between continuous ($M = 40.1, SD = 19.4$) and discrete ($M = 40.0, SD = 23.0$) conditions on the step of the winning bid. The condition of sphericity was not met for the block analysis ($\chi ^2 = .12, p = 0.04$); therefore, degrees of freedom were corrected using Greenhouse-Geisser estimates of sphericity ($\epsilon = .56$). There was no significant main effect ($F(2.23,22.25) = 1.448, p = 0.26, BF_{10} = 0.21$) of blocks on step. Following the statistically non-significant difference between blocks for both price and step of the winning bids, we continued analysis of the data collapsed across blocks. These results indicate that the average winning bid did *not* change across blocks in either of the step-rate conditions.

### Effect of starting price on winning bids

**Fig. 8:**
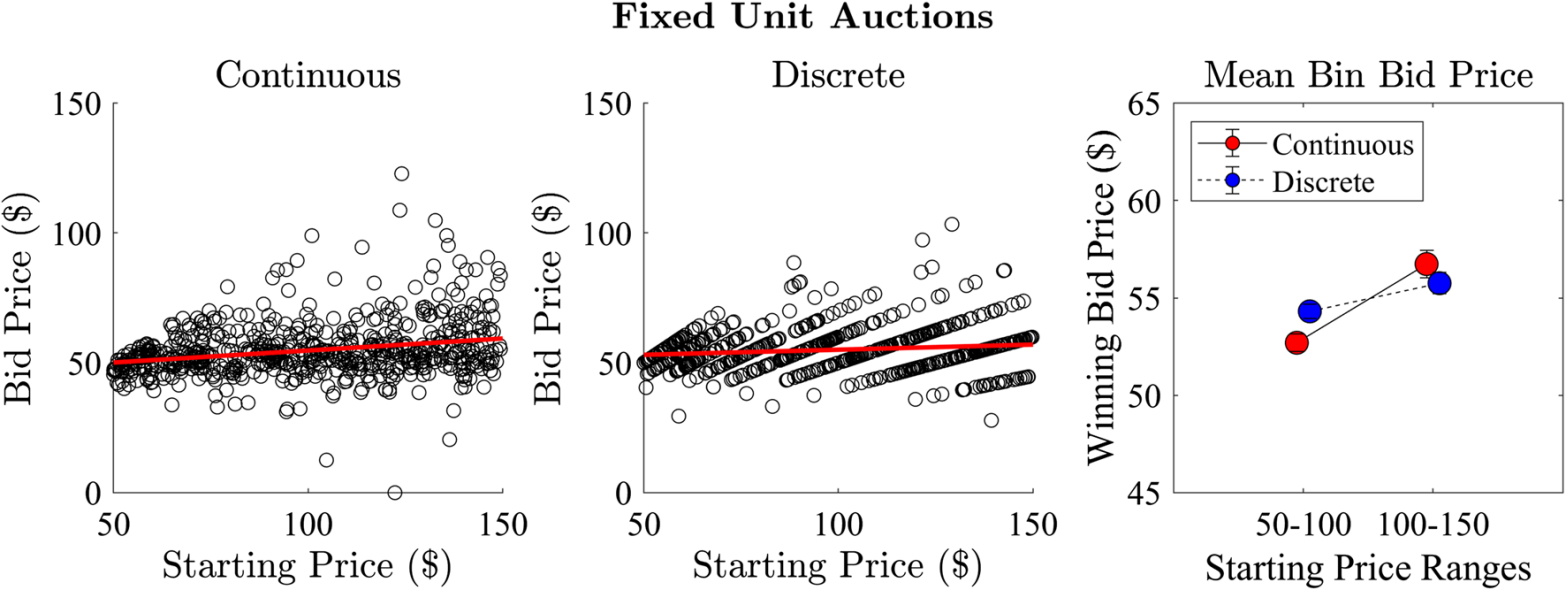
Experiment 1: relationship between auction starting price and the price of the winning bid for continuous (left) and discrete (middle) step conditions and the mean price of the winning bid for auctions separated into high and low starting price (right)

We assessed the relationship between the starting price and the price of the winning bid for both the continuous and discrete step conditions (Fig. 8). There was a significant positive correlation between starting price and bid price in the continuous ($$r = 0.251, R^2 = 0.063, p < 0.001$$) and discrete ($$r = 0.139, R^2 = 0.019, p < 0.001$$) conditions. We further assessed this relationship by separating the bid data into bins based on low ($50-100) and high ($100–150) starting prices. There was a significant difference between the high and low starting price auctions with strong evidence in the continuous ($$t(658) = -4.85, p < 0.001, BF_{10} = 7,392$$) and a significant difference in the discrete condition ($$t(658) = -2.22, p = 0.027$$, which was not confirmed by Bayes factor, $$BF_{10} = 0.96$$). We repeated the analysis for different bin sizes and found a similar trend (see Appendix A).

## Experiment 2: multi-unit

### Method

Experiment 2 was identical to Experiment 1 except that purchasable stock on each trial varied randomly between 50 and 150 units rather than being fixed at 100 units.

#### Participants

Thirty-three students ($$M_{\mathrm{age}} = 23.3, {\hbox {SD}}_{\mathrm{age}} = 4.0$$; 17 female, 16 male) from the University of Newcastle, Australia, who had not participated in Experiment 1, placed into 11 three-person groups. All participants reported English as their first language and had normal or corrected to normal vision, including colour vision. Participants were reimbursed with a $25 shopping voucher or course credit, depending on sign-up source.

#### Stimuli and design

Stimuli and design were identical to Experiment 1 with the exception of the varying value of available stock. The available stock was presented to participants as a red bar in the game interface, where size of the bar varied to reflect the available stock (i.e. a large amount of stock was represented by a larger bar).

### Results and discussion

Practice block data were removed from analysis. A summary of Experiment 2 results is presented in Table 2.

**Table 2:** Experiment 2 results summary

		Price		Step	
		*p*	${\hbox {BF}}_{10}$	*p*	${\hbox {BF}}_{10}$
Group (1–11)	Continuous	.95	< .001	.67	0.002
	Discrete	.99	< .001	.05	0.09
Continuous versus discrete	Means	.84	0.05	.05	0.28
Block (1–5)	Step rate	.78	0.21	.79	1.05
	Block	.36	0.13	.37	0.17

*Denotes significance

**Fig. 9:**
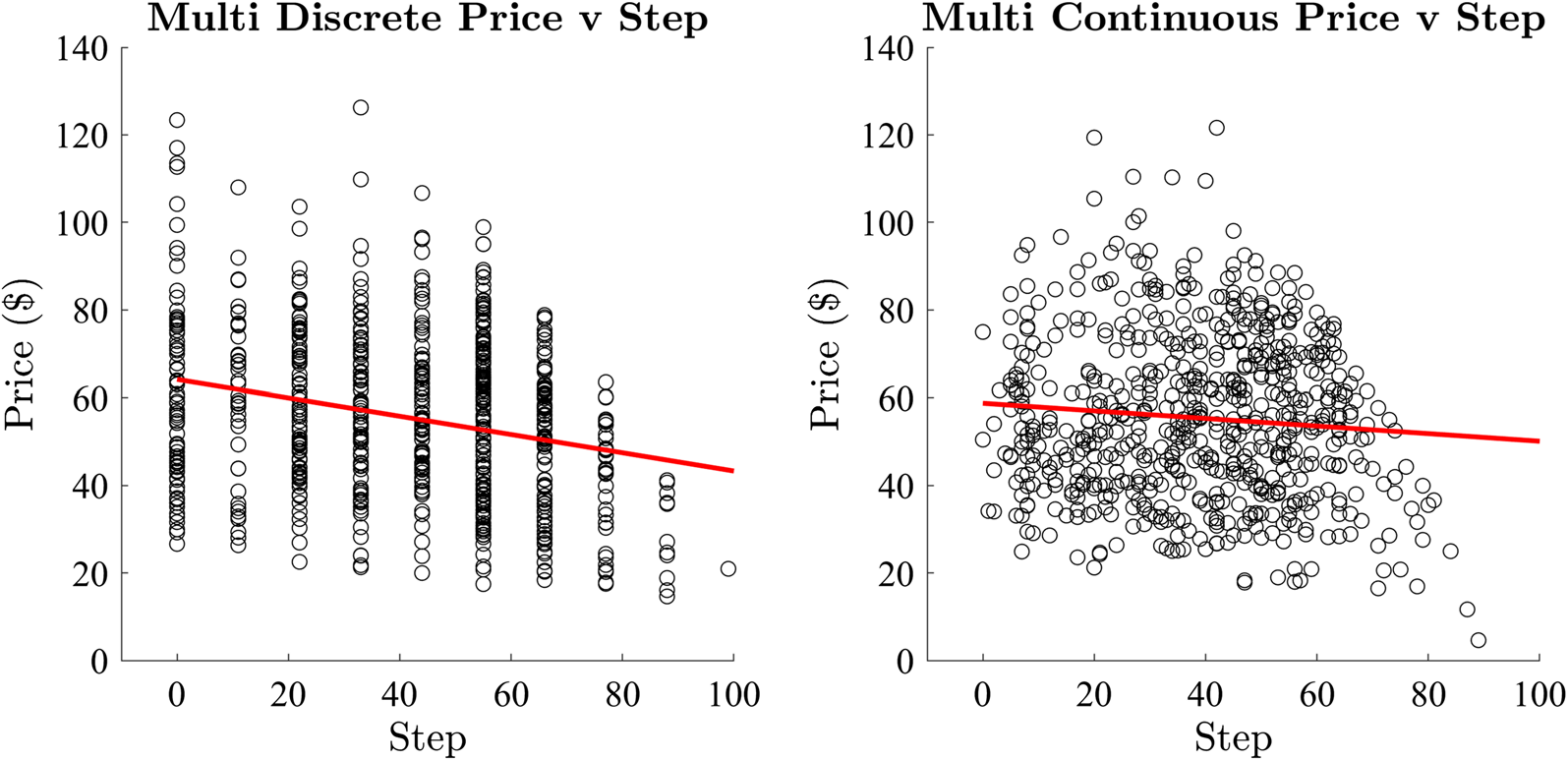
Exp 2: correlation of the price and step of winning bids

We calculated the correlation between the price and step of the winning bid in both the discrete and continuous conditions (Fig. 9). There was a significant moderate negative correlation between price and step in the discrete price-change condition ($r = -0.25, R^2 = 0.06, p < 0.001$). There was also a significant weak negative correlation between price and step in the continuous price-change condition ($r = -0.09, R^2 = 0.01, p = 0.03$). As in Experiment 1, we anticipated negative correlation, which is imperfect due to variability in starting price, possibly compounded by the additional variability in the total items for sale for each auction. We continue the examination of the data using both of these measures.

#### Effect of participant group

**Fig. 10:**
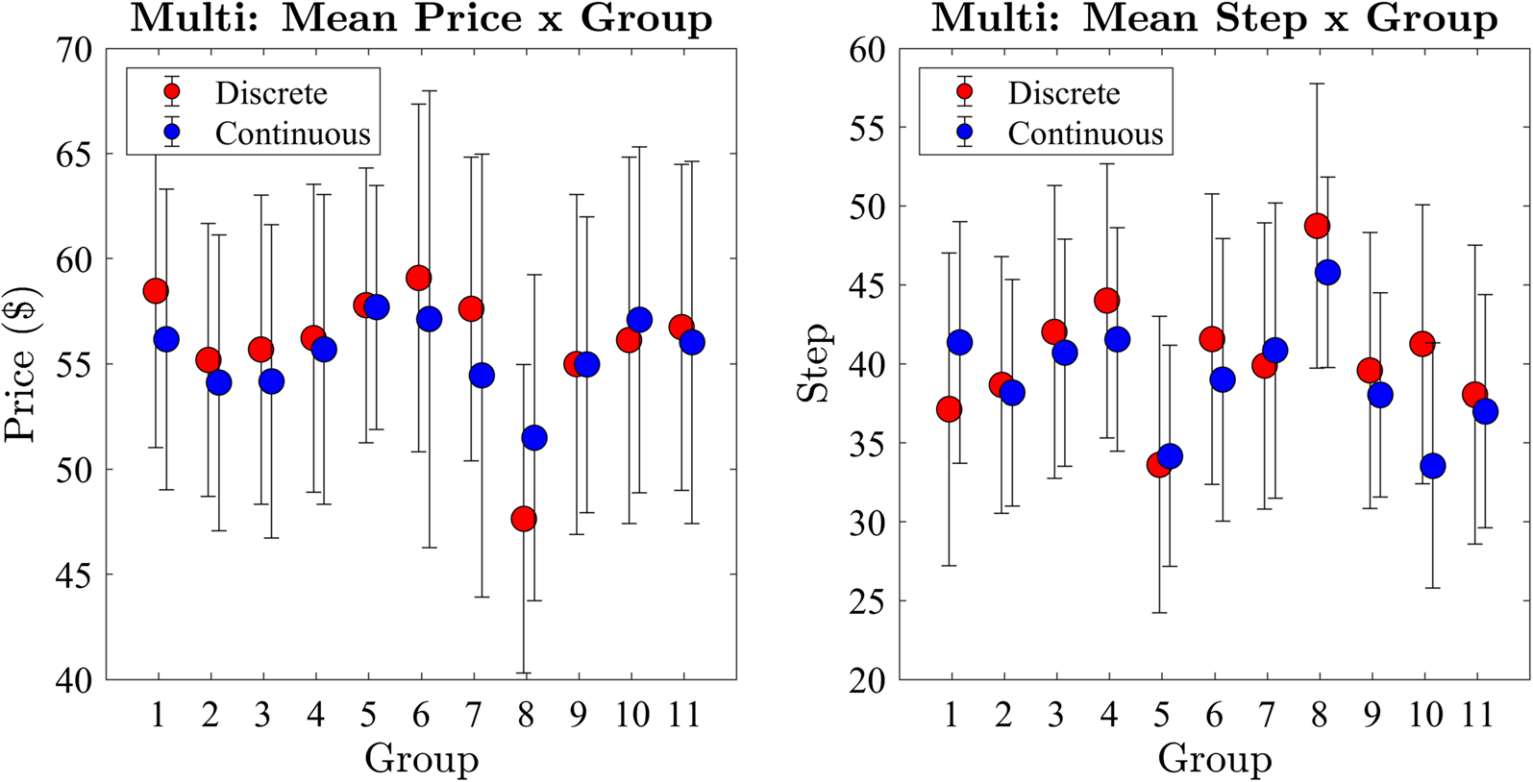
Exp 2: mean price and step per group

We analysed the effect of participant group on the price of the winning bid (Fig. 10) with separate one-way between subjects ANOVAs for the discrete and continuous price change conditions. There was no significant difference between groups on the price of the winning bid in the discrete ($F(10,649) = 0.26, p = 0.99, BF_{10} < 0.001$) and continuous conditions ($F(10,649) = 0.38, p = 0.95, BF_{10} < 0.001$).

There was a significant difference between groups on step of the winning bid for the discrete ($F(10,649) = 1.85, p = 0.05$) condition; however, there was no evidence supporting the null hypothesis ($BF_{10} = .09$). A post hoc analysis using the Bonferroni correction for multiple comparisons was conducted which did not identify any significantly different relationships between the participant groups. We found no significant effect on the step between groups in the continuous ($F(10,649) = 0.76, p = 0.67, BF_{10} = 0.002$) condition. As there was no substantial variation between the price and step of the winning bid between participant groups, we continued analysis with data collapsed across participant groups.

#### Discrete versus continuous price change

**Fig. 11:**
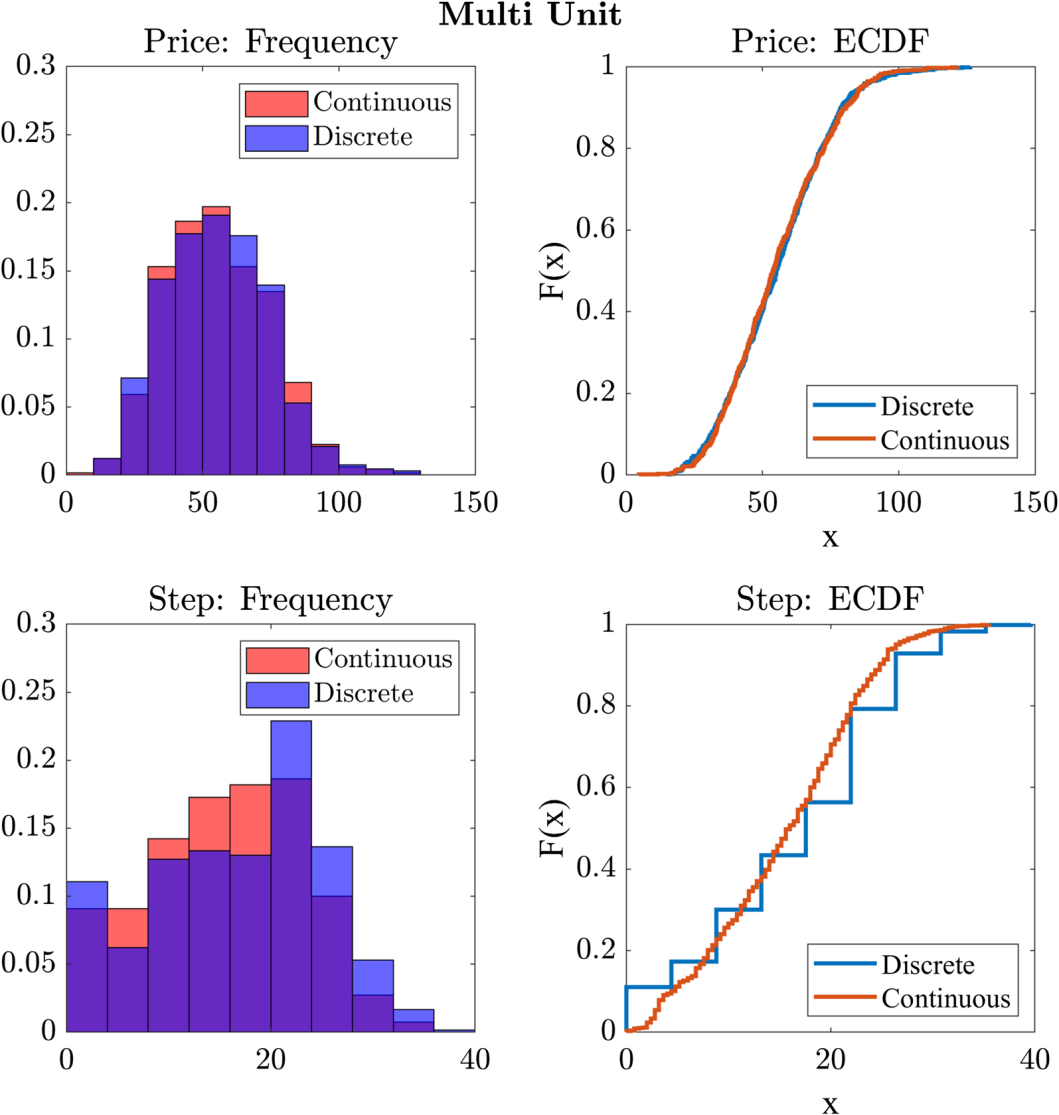
Exp 2: cumulative distribution functions of price and step

We then compared the price of the winning bid across the discrete and continuous step conditions using paired samples *t* tests as, unlike Experiment 1, all assumptions were met. We found no significant difference ($t = -0.2, p = 0.84, BF_{10} = 0.05$) between the continuous ($M = 55.4, SD = 18.75$) and discrete ($M = 55.6, SD = 19.0$) price change conditions on the price of the winning bid (Fig. 11).

We repeated the analysis with the step of the winning bid and found no significant difference, with moderate evidence for the null ($t = -1.94, p = 0.05, BF_{10} = 0.28$), between the continuous ($M = 38.8, SD = 18.6$) and discrete ($M = 40.9, SD = 23.2$) conditions. These findings indicate that there is no significant effect of the step-rates used on the price or timing of the winning bid in a competitive group computerised Dutch auction format.

#### Effect of block

**Fig. 12:**
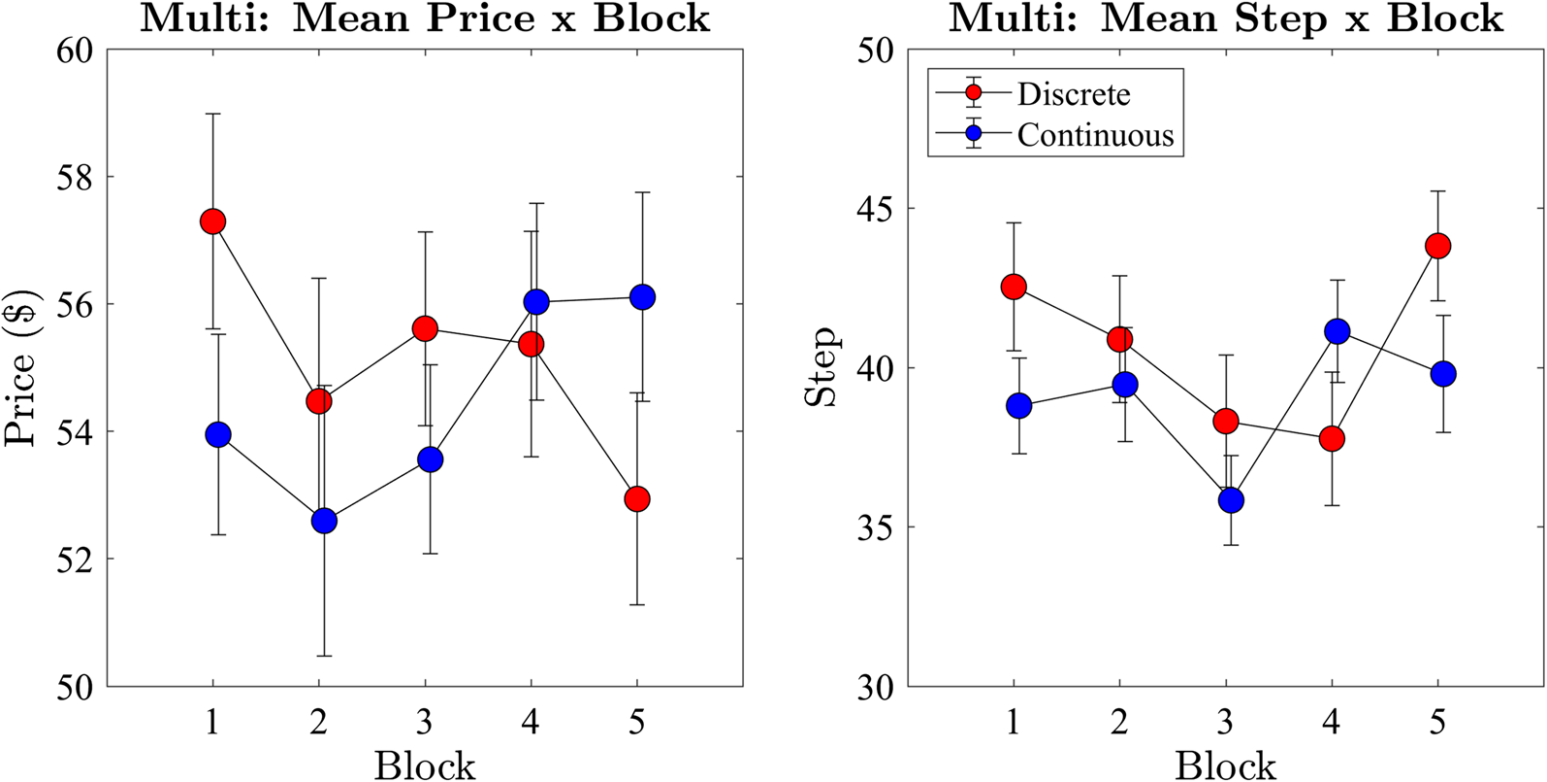
Exp 2: mean price and step of winning bids per block

We next examined the price and step of the winning bid across blocks (Fig. 12). Price of the winning bid data was analysed with a two-way ANOVA with within subjects factors condition (discrete and continuous) and block (1st-5th). There was no significant main effect ($F(1,10) = 0.08, p = 0.78, BF_{10} = 0.21$) on price between the continuous ($M = 55.4, SD = 18.8$) and discrete ($M = 55.6, SD = 19.0$) conditions. As with Experiment 1, the assumption of sphericity was violated for the analysis of blocks ($\chi ^2 = .09, p = 0.02$). We applied the Greenhouse-Geisser correction ($\epsilon = .57$) and found no significant main effect ($F(2.3,22.97) = 1.08, p = 0.36, BF_{10} = 0.13$) between blocks on the price of the winning bid.

We repeated the ANOVA with the step of the winning bid and found no significant difference ($F(1,10) = 4.28, p = 0.79, BF_{10} = 1.05$) of the step between the continuous ($M = 38.8, SD = 18.6$) and discrete ($M = 40.9, SD = 23.2$) conditions. There was also no main effect ($F(4,40) = 1.11, p = 0.37, BF_{10} = 0.17$) between blocks, suggesting winning bids did not change across blocks in either of the step-rate conditions as participants gained auction experience.

### Effect of starting price on winning bids

**Fig. 13:**
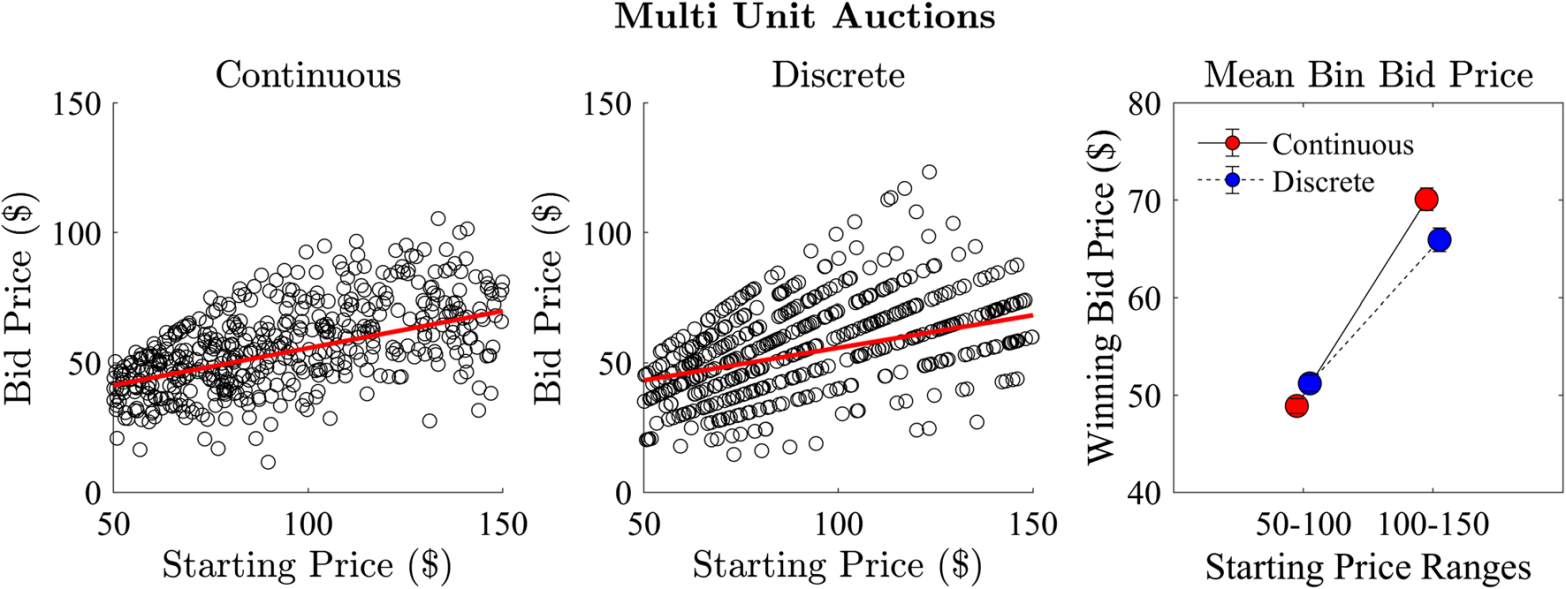
Experiment 2. Relationship between auction starting price and the price of the winning bid in continuous (left) and discrete (middle) step conditions. The mean price of the winning bid for auctions was separated into low and high starting price (right) for both step conditions

Finally, as in Experiment 1, we assessed the relationship between the starting price and the price of the winning bid for both the continuous and discrete step conditions (see Fig. 13). There was a significant positive correlation between starting price and bid price in the continuous ($$r = 0.659, R^2 = 0.434, p < 0.001$$) and discrete ($$r = 0.539, R^2 = 0.29, p < 0.001$$) conditions. We then separated the bid data into low ($50–100) and high ($100–150) starting prices and found a significant difference between the high and low starting price auctions with strong evidence in the continuous ($$t(658) = -15.706, p < 0.001, Log(BF_{10}) = 101.058$$) and discrete condition ($$t(658) = -9.675, p < 0.001, Log(BF_{10}) = 40.628$$).

## General discussion

We developed a novel platform for testing competitive decision making in a simulated Dutch auction. The platform allows manipulation of three fundamental design features—start price, rate of price change and size of price change [see Cox et al. (1982)] and records the winning-bid price and winning-bid time step across the various conditions. We briefly recap the main findings and then present a novel adaptation of Kahneman and Tversky’s prospect theory (1979, 1992) to account for qualitative patterns in the data, including the continuous and discrete step outcomes.

In both Experiments 1 and 2, there was no significant difference in the price or step (i.e. time at which participants bid) of the winning bid between the discrete price change and continuous price change conditions. These outcomes were supported by Bayes factor analysis which allowed the assessment of null effects, thereby overcoming limitations of frequentist tests. We also found no significant difference in either the Price or Step of the winning bid across testing blocks in either experiment.

### Effect of price change patterns on bidding behaviours

The findings of the current study suggest there was no significant difference in the Price or Step of the winning bid between discrete and continuous price-change conditions. This supports the findings of Katok and Kwasnica (2008) where the overall duration of the auction, rather than differences in patterns of price changes, influenced bidding behaviour. Katok and Kwasnica (2008) found that Dutch auctions with slow price changes (i.e. longer time intervals between price decreases) resulted in higher priced winning bids when compared to Dutch auctions with fast price changes (i.e. small time intervals between price decreases). However, their manipulations to the speed of price change resulted in changes in the overall duration of the auctions. The slow price change auction ran for a maximum of 10 min, while the fast price change auction ran for a maximum of 20 s. It is also worth noting that Katok and Kwasnica (2008) used financial incentives for both winning auctions and for finishing the task. They acknowledged that participants may have bid earlier, thus raising the price of bids, to end the auction and receive their payout earlier. In the current study, we maintained overall duration across the discrete and continuous price change conditions, so were able to focus on the effect of different patterns of price changes while controlling for the overall duration of the auction. With no difference observed in bidding behaviours between the discrete and continuous condition, it is possible that the overall duration of the auction affects bidding behaviours rather than the pattern of price changes.

Our findings support a different aspect of Katok and Kwasnica (2008) theory—that difference in bidding behaviours is caused by bidders considering time as a valuable resource, resulting in a trade-off between time saved and price. In the current study, the overall duration of the auctions was held constant across price conditions (fast, slow), so there was no need for bidders to trade-off between time saved and price, and without financial incentive for task completion there was no direct gain for ending auctions prematurely. This resulted in similar Price and Step of winning bids across the discrete and continuous price-change conditions.

This outcome may have been affected by the short duration of individual auction trials used in the current study. Each individual auction-trial in both the discrete and continuous price-change conditions ran for a maximum of 5 s. This short duration of individual auction [relative to Katok and Kwasnica (2008)] may have not allowed for the perceptual differences in the different patterns of price changes to visibly affect bidding behaviour. Future research may benefit from utilisation of our platform to examine the effect of different patterns of price changes over longer-duration auctions, where the perceptual difference is more apparent to the bidders.

### Consideration of hypothetical bidding in competitive decision-making

Next, we consider the potential effect of hypothetical funds on bidding behaviour. Without financial incentives to motivate participants to engage in real-world behaviours, it is possible our non-significant results may be an outcome of this design feature. However, in an extensive review of incentivised versus non-incentivised experiments in economics and psychology, Camerer and Hogarth (1999) concluded that incentives are less likely to affect mean performance in games, auctions, and risky choice tasks; however, incentives can reduce response variance. From a psychological perspective on participant effort, Erkal et al. (2018) found that non-monetary incentives like task enjoyment, desire to perform well and, importantly, competitiveness motivated participants to exert an equally high effort when compared to the same incentivised two-player task. Nonetheless, there are also studies suggesting different behaviour in incentivised versus non-incentivised tasks, and this could be tested in future investigations of Dutch auction bidding behaviour.

### Effect of learning across blocks on bidding behaviours

Garvin and Kagel (1994) found that inexperienced bidders initially bid earlier at high prices, resulting in the winner’s curse (the tendency to bid higher than the value of the auctioned item). However, with experience these participants could adjust their bidding behaviours, reducing the magnitude of the winner’s curse.

We examined whether exposure to the auction format, and interaction with other competitors, would affect participant bids by assessing the group’s mean price and step of the winning bids across the five blocks (12 trials per block) of a testing session. We found that the mean price and step of winning bids in Dutch auctions with either a fixed number of units for sale (Experiment 1) or varying units for sale (Experiment 2) was not significantly different. These results may arise from participants beginning the experiment with a near optimal or good estimation of item value based on experimental design features. For example, in each block participants were asked to use a fixed allocation of funds to purchase stock to fill their fixed size virtual warehouse. By providing each participant with $250 to spend and a warehouse capacity of 500 units, a participant had to win a total of 5 trials (i.e. auctions) in Experiment 1 or an average of 5 trials in Experiment 2 at an average of $50 per bid to successfully fill their warehouse. Our results suggest that participant triads may have already determined a bidding price strategy, averaging a winning bid of around $55 across all blocks in both conditions. Alternatively, the optimal use of funds would be through five $50 bids. While the competitive environment may drive the final bids up from this optimal price, Turocy et al. (2015) have also found that some participants will not update their bidding strategy. The authors found that only some participants changed bid price across an auction while others exhibited winner’s curse bidding but did not utilise information to update their bidding strategy to reach an optimal bid-price. Whether all group members identified an equivalent bidding strategy from the auction design or some individuals adapted their bidding strategies and others did not, our results indicate that the price of winning bids within a group competitive bidding environment are not affected by exposure to other competitors or the auction format.

### Consideration of certainty and utility on bidding behaviour: a prospect theory account

A fundamental feature of the Dutch auction is the certainty of winning (and losing)—the bidder who is first to bid wins the available item with certainty (Turocy et al. 2007). However, this is only true to the extent that no other player had yet placed a bid at that point in time. With passing time, there is an increasing likelihood that other stakeholders will place a bid, reducing the chances the item is still available. Our experiments required participants to trade off between the certainty of winning the bid, which decreases over the time course of the auction trial, and the price they are willing to pay for the available items (which also goes down).

Balancing risk, certainty, and value (alternatively, utility) is a standard feature in theories of economic decision-making. One of the most influential theories is prospect theory (Tversky and Kahneman 1992), which is commonly applied to single-player scenarios with standard choices about gambles such as “would you rather take a certain gain of $100, or a 50–50 chance of winning $200?”. We developed a new adaptation of prospect theory to account for multiple players’ bidding behaviour in Dutch auctions.

Prospect theory has been widely considered in ascending price auction formats; however, it has not been applied to Dutch auctions in a quantitative manner. For example, Kuruzovich (2012) discussed processes by which bidders increase their valuation of items through interaction with an online auction mechanism (but not necessarily *Dutch* Auctions). They argued from a prospect theory perspective that Dutch auctions present the individual with a different decisional frame compared to other auction formats, as Dutch auctions begin at a high price point and decreases, rather than start at a small value that increases. By commencing auctions at a higher value, the auctioneer changes the external framing of the choice, which should theoretically result in a higher bid and overall revenue from the auction. Fu et al. (2018) used regret theory to claim that participants will bid earlier in Dutch auctions to avoid feelings of regret as they are *loss averse*, a central prediction of prospect theory. They hypothesised that higher starting prices in a Dutch auction would increase the perceived valuation of items, resulting in higher bids as individuals become more loss averse. Similarly, Dodonova and Khoroshilov (2009) argue that the endowment effect, another example of loss aversion where individuals place higher value on items they already own, should be seen in the reserve prices set in a Dutch auction by sellers. While prospect theory has provided a sound theoretical framework to develop hypotheses and interpret results, we are not aware of any quantitative adaptation of prospect theory to Dutch auction decision making.

Our approach was to extend prospect theory in the time domain, by assuming that each player makes a *sequence* of choices during the auction. These repeated choices are all binary decisions: each time, the player must decide whether to bid immediately, or to wait just a few moments longer. Bidding immediately—given the auction is still running—is a prospect comparable to the “certain $100” above, in that the player will be guaranteed to win the auction, so they know both the gain (the product for sale) and the loss (the current price) associated with the choice. Waiting is a prospect comparable to the risky option above: the player must estimate the risk associated with waiting longer, the probability that another player will bid in the next few moments. Prospect theory (Tversky and Kahneman 1992) provides a well-established way to predict the choices of people faced with decisions between these options. In prospect theory, the choice relies on the perceived value of the item for sale (its “utility” to the bidder), the perceived value of the loss of money (the utility loss associated with paying the price), and the uncertainty (the probability of being beaten by another player in the next few moments).

We operationalise the prospect theory model as follows. Suppose *t* represents the time in the auction, and *C*(*t*) represents the selling price, or cost, at time *t* (in our experiments, *C* is a linear function). Suppose also that the perceived value of the product to the bidder is *P* and that the chance of another player bidding (and ending the auction) in the next few moments between time *t* and time $t+dt$ is *r*. Prospect theory converts the costs to subjective utilities via a simple power function, *U*, and converts the probability to a subjective weighted probability via a sigmoid function, $\pi$. The details of those functions are standardised in prospect theory and are reproduced below as well. The choice to buy now has no risk, and so has a net utility of $U(P)+U(-C(t))$. The net utility of buying the product in a few moments is better, because the price will be reduced, $U(P)+U(-C(t+dt))$, but this outcome must be weighed against the probability of being beaten by another player, which has zero gain and loss. This makes the overall weighted utility associated with waiting $\pi (1-r)(U(P)+U(-C(t)))$.

We assume standard forms for the utility and probability weighting functions. Utility (*U*) is a power function of price (*x*), with different behaviour on losses (negative prices) than gains:

$$\begin{aligned} U(x,\alpha ,\beta ) \ = \ x^{\alpha } \ \text {if} \ x>0, \\ -\lambda (-x)^{\beta } \ \text {if} \ x<0 \end{aligned}$$

The probability weighting function ($\pi$) is defined below by two parameters, to allow separate weights for gains and losses, where we replace $\gamma$ with $\delta$ for losses:

$$\begin{aligned} \pi (p,\gamma ) = (p^{\gamma }/(p^{\gamma } + -p^{\gamma }))^{1/\gamma } \end{aligned}$$

The above defines a weighted utility for each of the two choices— bidding now, at time *t* versus waiting to bid at time $t+dt$. Prospect theory converts these utilities into a probability of choice using a “softmax” rule. Suppose we let $U_t$ represent the weighted utility of bidding at time *t* and $U_{t+dt}$ represent the weighted utility of bidding at time $t+dt$. Then:

$$\begin{aligned} \mathrm {Pr}(\mathrm {bid} \, \mathrm {now}) = \frac{e^{-cU_t}}{e^{-cU_t} + e^{-cU_{t+dt}}} \end{aligned}$$

Parameter *c* represents a player’s sensitivity to differences in value. When *c* is large, the player will almost certainly choose the option with the larger weighted utility, even if the differences between options are very small. When *c* is small, the player sometimes chooses randomly, selecting the lower-utility option on some occasions.

The model relies on the player having some estimate of the probability that one of the other players will bid in the next few moments, *r*. We operationalise this by assuming that the player maintains some representation of the bidding-time distributions of the other players. If this distribution is assumed to be identical for the two other players, with density *f*(*t*) and cumulative distribution *F*(*t*), then it is simple to show that $r= 1-(1-(F(t+dt)-F(t)))^2$.^2^ For the simulations below, we made the simple assumption that each player estimated the other players’ bidding times as normally distributed, $N(\mu ,\sigma )$.

**Fig. 14:**
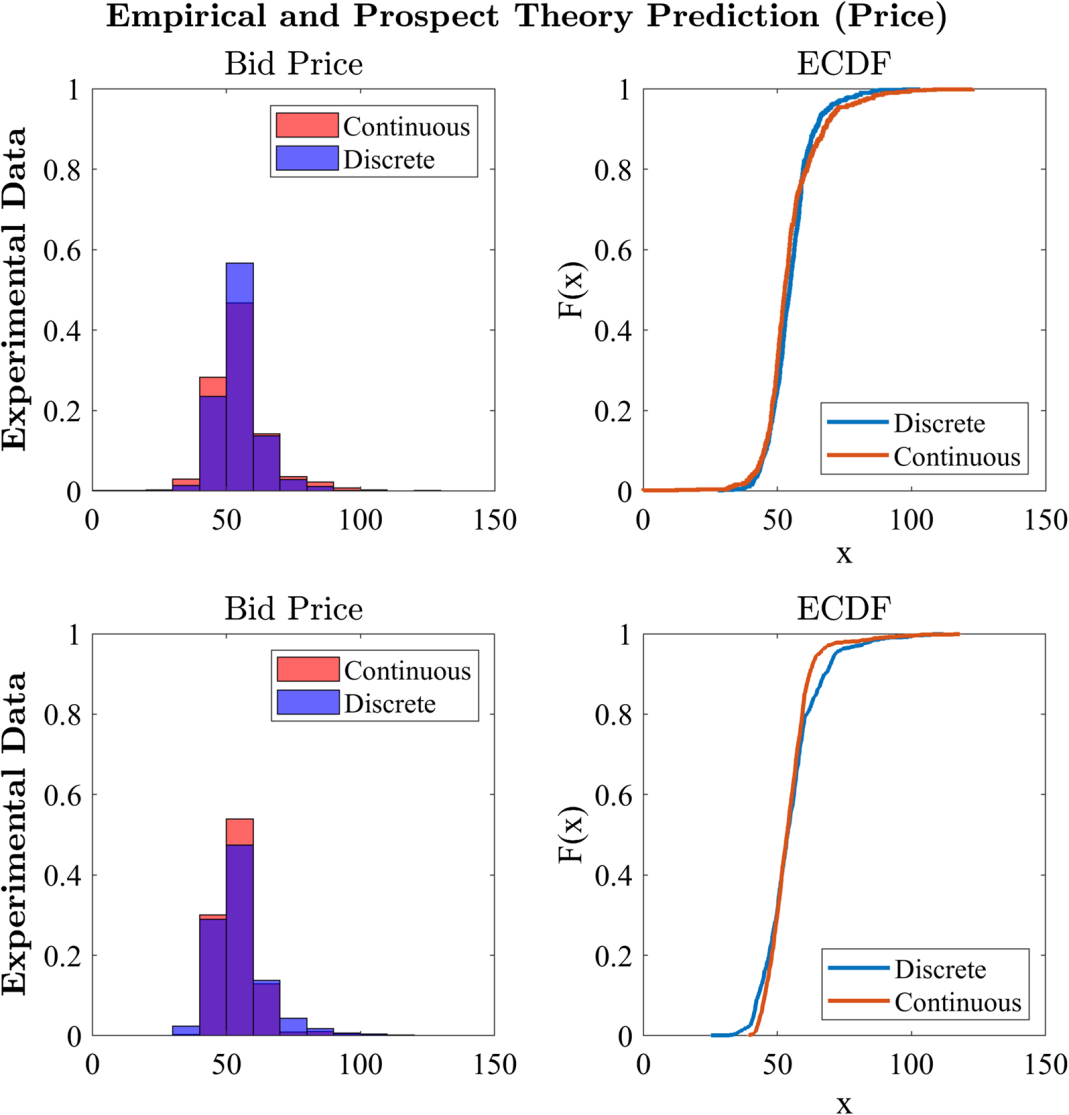
Empirical data (top) and prospect theory predictions (bottom) of price of the winning bid

The above functions describe the probability of a single player making a bid in the next few moments. This is a hazard function, which can be converted to a probability density function (say, *g*) and associated cumulative distribution function (say, *G*) by standard transformations. However, the empirical distributions collected in our experiment depict the price and time of the winning bids, across all three players (e.g. player 1 could have won the first auction, but another player could have won the next auction in the block). To link our theoretical predictions with the data, we must infer from the model the empirical distribution of bidding prices in all auctions, by marginalising over *all* players—not just a single player. This reflects the summary distributions shown in figures such as Fig. 14. Thus, the final step is to let the model define the behaviour of three concurrent players and to derive from this the distribution of the minimum bidding times. This is done by taking the minimum over the three players, which has cumulative distribution $1-(1-G(t))^3$.

We now provide a sufficiency proof for the model, by demonstrating that it is capable of generating bidding patterns similar to those observed in empirical data (at least for some parameter combinations—a sufficiency proof). We simulated three-player group bidding data over 1000 fixed unit auctions. We set the behaviour of the auction price clock, *C*(*t*) to be randomly sampled from a uniform distribution between $80 and $120 and decrease to $0 linearly over 5 s.

We set the time step to $$dt=0.5$$, matching the discrete condition or to $$dt=0.05$$, matching the continuous condition. Figure 14 illustrates empirical distributions of Experiment 1 data (top, similar to the distributions plotted in Fig. 6) and the simulated bid data produced by the model predictions of the winning bid price. We used parameter values taken from the best fits determined by Tversky and Kahneman (1992): $$\alpha =0.88, \beta = 0.88, \lambda = 2.25, \gamma = 0.61, \Delta = 0.69$$. For the other parameters, we investigated numerous combinations and plot here one example set. The mean ($$\mu = 2.01$$) and standard deviation ($$\sigma = 0.97$$) of the estimated bid time were derived from the empirical data as the mean and standard deviation of group bidding time and remained fixed for the continuous and discrete estimates but, given the difference in step size and associated price difference between steps, the perceived value (*V*) of goods and sensitivity (*c*) parameters differed between the continuous ($$V = 1.27, c = 2.1$$) and discrete ($$V = 1.43, c = 0.3$$). These parameter combinations, and others, can generate distributions of winning bid price similar to the empirical data, as can be seen in Fig. 14. To make the model accurately predict both the average bid and the detailed distribution of bids, we had to simulate the model using a range of starting prices ($80 to $120) smaller than the range used in the experiment ($50 to $150). This represents an interesting theoretical observation that participants were less sensitive to changes in the stated cost (i.e. the auction-clock price) than the most straightforward application of prospect theory would predict. This may suggest a reference point effect. We constrained our model to use a very standard account, in which all prospects were treated as changes from a zero-dollar reference point. However, our players may have treated the prospects differently, as gains and losses relative to their current circumstances. With nonzero reference points, the nonlinear effects of the weighting and utility functions are changed, leading to effects such range compression and expansion (e.g. the range of [0.5, 1] is the same as the range [10.5, 11], but their ranges are very different after log scaling, such as in a utility function). This represents an interesting insight that may be investigated in future research.

### Effect of starting price on winning bids

The model produces predicted distributions of bids that are similar to the empirical data, for some parameter values. We wanted to test more specific predictions of the model by examining predictions of auction starting price, for which prospect theory can be used to make predictions in Dutch auctions (see Kuruzovich 2012). There is a lack of consensus in the general auction literature (not necessarily Dutch auctions) concerning the effect of starting price. Some evidence shows lower starting prices lead to higher bids (e.g. Ku et al. 2006), while others find evidence to the contrary (lower start prices lead to lower bid, e.g. Ariely and Simonson 2003). Ku et al. (2006) analyzed how low starting prices attract an entry of new bidders and affect final prices. Based on eBay field data and survey experiments, the authors found low starting prices attracted more bidders and thereby result in higher final prices. Walley and Fortin (2005) confirmed in their controlled field experiment that lower starting prices increase the number of bidders and eventually final prices. Ariely and Simonson (2003) also confirm the results of Ku et al. (2006), stating that low starting prices attract more bidders, with data from a controlled field experiment on eBay. However, and in contrast to Ku et al. (2006), Ariely and Simonson (2003) find that these auctions yield on average fewer bids and lower final prices. The authors argue that this can be explained by an anchoring effect: starting prices may serve as a value signal for bidders, with higher starting prices indicating a higher product value. Although this effect has not been examined in Dutch auctions, Kuruzovich (2012) argued that because Dutch auctions start at a high price and decrease they should theoretically produce higher revenue compared to ascending price auctions; however, this was not empirically tested.

In both Experiments 1 and 2, there was a positive correlation between starting price and price of winning bids, in both fixed- and multi-unit Dutch auctions. We found that the price of winning bids was significantly different when separated into high and low starting price bins. Here, we implement the same analysis on the simulated data from our prospect theory based model and show the model predicts this qualitative pattern.

**Fig. 15:**
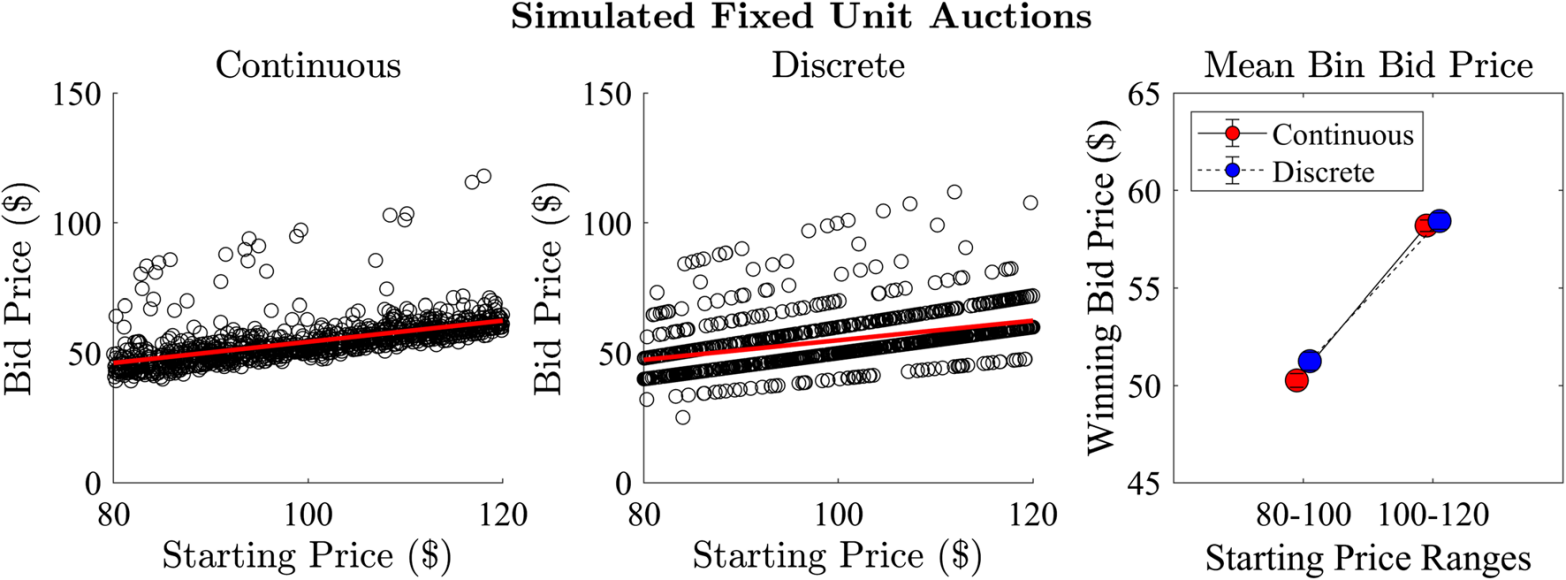
Positive relationship between auction starting price and winning bid for continuous (left) and discrete (middle) conditions in model simulated Dutch auction group bidding. Mean bid price across low and high auction start prices (right) in the model-simulated data displays the same increase pattern in bid prices as the empirical data (cf. Fig. 8)

Figure 15 depicts the positive relationship between the auction starting price and the price of the winning bid for model-simulated data. The correlation was significant in both the continuous ($r = 0.574, R^2 = 0.330, p < 0.001$) and discrete ($r = 0.417, R^2 = 0.174, p < 0.001$) conditions.

We further assessed the model-simulated winning bid prices when bids were separated into auctions commencing at a low ($80–$100) or high ($100–$120) price (Fig. 15, right). We found a significant difference between the low and high starting price auctions with strong evidence in the continuous ($$t(998) = -17.1, p < 0.001, Log(BF_{10}) = 124.56$$) and discrete ($$t(998) = -11.47, p < 0.001 , Log(BF_{10}) = 58.5$$) conditions. We explored this relationship across different bin sizes in Appendix A and found it to be a robust effect in both data sets.

These results suggest starting price is related to the price of the winning bid in fixed unit Dutch auctions under continuous and discrete step-rates. This finding is important for both theoretical and practical reasons. From a theoretical perspective, it allows to compare the empirical pattern with model predictions. Practically, starting price of an auction is a design choice of the auctioneer or market designer. If they start the auction with too high a price, they might waste valuable time, which is especially critical when selling perishable items (as is the case in most Dutch auctions). If they start too low, they might miss out on additional revenue as predicted by both our data and model.

### Conclusion and future directions

The current study aimed to develop a computerised platform for Dutch auctions and test how different design parameters affect the decision-making processes involved in this competitive group context. Results from Experiments 1 (fixed item quantity) and 2 (variable item quantity) showed no significant effect for different patterns of price changes on the price or time-step of the winning bid. There was no difference in the price or step of the winning bid between testing blocks. This suggests that participants either (1) began with a good estimate of item value or (2) did not change their bidding behaviour through experience.

Empirical data were collected in-lab. This had limited the sample size, as the scheduling of multiple participants to concurrent testing is non-trivial. Future studies could employ online testing to obtain larger sample. However, in-lab testing rewarded the study with very real and vivid group context. In post testing interviews, some participants reported they were excited by the competitive nature of the task. Future research may look into physiological measures, to assess arousal and how it affects bidding behaviour (a-la Malhotra et al. 2008).

In conclusion, this paper offers theoretical and practical contributions. On the theoretical side, we developed an adaptation of prospect theory that can account for bidding behaviour in Dutch auction. Our results also have practical implications: to the extent that they can be generalised, they suggest that (1) for a given rate of price-change, the pattern of change (small decrements over many steps or large decrements over few steps) has little-to-no effect of bidders’ behaviour, and (2) that increasing the starting price of a fixed unit Dutch auction results in an increase in the price of bids. The model successfully captured both patterns. Our testing platform provides an exciting avenue for future research into biding behaviour. The Dutch auction allows to investigate the way multiple participants within a group balance risk (of missing the bid) and cost (bidding price) and serves as an ideal context for the study of competitive decision making.

## Figures and Tables

**Fig. 16:**
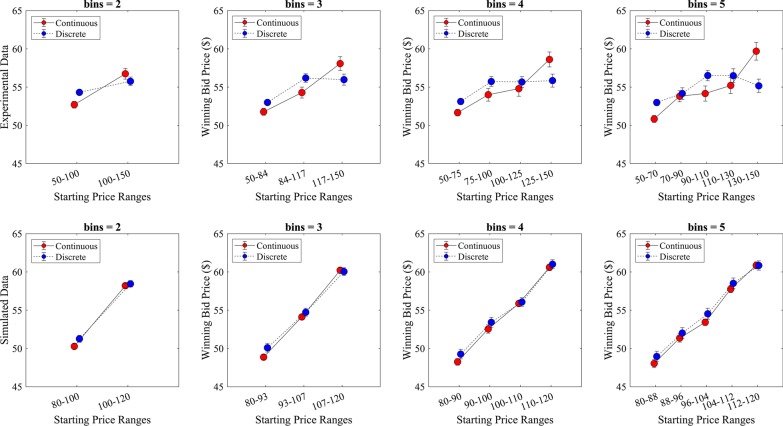
Experimental Data (top) compared to model simulated data (bottom) for both the continuous (red) and discrete (blue) conditions

## Data Availability

The datasets, analysis and experiment platform codes of the current study are available at https://osf.io/jyv3u/.

## Starting price bin sizes

Here we present the price of the winning bid in fixed-unit Dutch auctions (Experiment 1) for different starting price bin ranges. Figure 16 shows an increase in the price of the winning bid with the increase in auction starting price over different starting price bin sizes. This effect is captured by the model, albeit at an increased magnitude.

We assessed the mean difference in winning bid for different bin numbers in both the continuous (Table 3) and discrete conditions Table 4) in the fixed unit auction (Experiment 1).

**Table 3:** Summary of ANOVA results—fixed continuous Price

Cases	Sum of squares	*df*	Mean square	*F*	*p*	${\hbox {BF}}_{10}$
Bins-2	2692.746	1.000	2692.746	23.563	$< 0.001$	7 392
Residual	75195.163	658.000	114.278			
Bins-3	4570.596	2.000	2285.298	20.479	$< 0.001$	3.632e+6
Residual	73317.312	657.000	111.594			
Bins-4	4342.039	3.000	1447.346	12.910	$< 0.001$	270 450
Residual	73545.869	656.000	112.113			
Bins-5	5516.367	4.000	1379.092	12.481	$< 0.001$	9.138e+6
Residual	72371.541	655.000	110.491

**Table 4:** Summary of ANOVA results—fixed discrete Price

Cases	Sum of squares	*df*	Mean square	*F*	*p*	${\hbox {BF}}_{10}$
Bins-2	342.538	1.000	342.538	4.914	0.027	0.956
Residual	45869.437	658.000	69.710			
Bins-3	1447.815	2.000	723.907	10.625	$< 0.001$	399.75
Residual	44764.161	657.000	68.134			
Bins-4	942.638	3.000	314.213	4.553	0.004	2.99
Residual	45269.337	656.000	69.008			
Bins-5	1314.789	4.000	328.697	4.795	$< 0.001$	11.7
Residual	44897.187	655.000	68.545			

## Footnotes

1 In addition, players could also be presented with information about the warehouse status of their competitors, but in the present experiments this feature was turned off, so that kind of knowledge was implicit.

2 Note that the distribution f need not have a total mass of 1 because the decision-maker may believe that it is not certain another player will buy at all. Also, we could allow the decision-maker to have separate beliefs about the distributions for each of the other players. This would then allow f, F to be defined as the distribution of maxima of the individual player’s distributions.
